# Microbiota enterotoxigenic *Bacteroides fragilis*-secreted BFT-1 promotes breast cancer cell stemness and chemoresistance through its functional receptor NOD1

**DOI:** 10.1093/procel/pwae005

**Published:** 2024-03-04

**Authors:** Wei Ma, Lu Zhang, Weilong Chen, Zhaoxia Chang, Juchuanli Tu, Yuanyuan Qin, Yuwen Yao, Mengxue Dong, Jiajun Ding, Siqin Li, Fengkai Li, Qiaodan Deng, Yifei Yang, Tingting Feng, Fanrong Zhang, Xiying Shao, Xueyan He, Lixing Zhang, Guohong Hu, Quentin Liu, Yi-Zhou Jiang, Shu Zhu, Zhi Xiao, Dan Su, Tong Liu, Suling Liu

**Affiliations:** Fudan University Shanghai Cancer Center & Institutes of Biomedical Sciences, State Key Laboratory of Genetic Engineering, Cancer Institutes, Department of Oncology, Key Laboratory of Breast Cancer in Shanghai, The Shanghai Key Laboratory of Medical Epigenetics, Shanghai Key Laboratory of Radiation Oncology, The International Co-laboratory of Medical Epigenetics and Metabolism, Ministry of Science and Technology, Shanghai Medical College, Fudan University, Shanghai 200032, China; Fudan University Shanghai Cancer Center & Institutes of Biomedical Sciences, State Key Laboratory of Genetic Engineering, Cancer Institutes, Department of Oncology, Key Laboratory of Breast Cancer in Shanghai, The Shanghai Key Laboratory of Medical Epigenetics, Shanghai Key Laboratory of Radiation Oncology, The International Co-laboratory of Medical Epigenetics and Metabolism, Ministry of Science and Technology, Shanghai Medical College, Fudan University, Shanghai 200032, China; Fudan University Shanghai Cancer Center & Institutes of Biomedical Sciences, State Key Laboratory of Genetic Engineering, Cancer Institutes, Department of Oncology, Key Laboratory of Breast Cancer in Shanghai, The Shanghai Key Laboratory of Medical Epigenetics, Shanghai Key Laboratory of Radiation Oncology, The International Co-laboratory of Medical Epigenetics and Metabolism, Ministry of Science and Technology, Shanghai Medical College, Fudan University, Shanghai 200032, China; Intelligent Pathology Institute and Department of Pathology, The First Affiliated Hospital of USTC, Division of Life Sciences and Medicine, University of Science and Technology of China, Hefei 230071, China; Fudan University Shanghai Cancer Center & Institutes of Biomedical Sciences, State Key Laboratory of Genetic Engineering, Cancer Institutes, Department of Oncology, Key Laboratory of Breast Cancer in Shanghai, The Shanghai Key Laboratory of Medical Epigenetics, Shanghai Key Laboratory of Radiation Oncology, The International Co-laboratory of Medical Epigenetics and Metabolism, Ministry of Science and Technology, Shanghai Medical College, Fudan University, Shanghai 200032, China; Fudan University Shanghai Cancer Center & Institutes of Biomedical Sciences, State Key Laboratory of Genetic Engineering, Cancer Institutes, Department of Oncology, Key Laboratory of Breast Cancer in Shanghai, The Shanghai Key Laboratory of Medical Epigenetics, Shanghai Key Laboratory of Radiation Oncology, The International Co-laboratory of Medical Epigenetics and Metabolism, Ministry of Science and Technology, Shanghai Medical College, Fudan University, Shanghai 200032, China; Fudan University Shanghai Cancer Center & Institutes of Biomedical Sciences, State Key Laboratory of Genetic Engineering, Cancer Institutes, Department of Oncology, Key Laboratory of Breast Cancer in Shanghai, The Shanghai Key Laboratory of Medical Epigenetics, Shanghai Key Laboratory of Radiation Oncology, The International Co-laboratory of Medical Epigenetics and Metabolism, Ministry of Science and Technology, Shanghai Medical College, Fudan University, Shanghai 200032, China; Intelligent Pathology Institute and Department of Pathology, The First Affiliated Hospital of USTC, Division of Life Sciences and Medicine, University of Science and Technology of China, Hefei 230071, China; Fudan University Shanghai Cancer Center & Institutes of Biomedical Sciences, State Key Laboratory of Genetic Engineering, Cancer Institutes, Department of Oncology, Key Laboratory of Breast Cancer in Shanghai, The Shanghai Key Laboratory of Medical Epigenetics, Shanghai Key Laboratory of Radiation Oncology, The International Co-laboratory of Medical Epigenetics and Metabolism, Ministry of Science and Technology, Shanghai Medical College, Fudan University, Shanghai 200032, China; Fudan University Shanghai Cancer Center & Institutes of Biomedical Sciences, State Key Laboratory of Genetic Engineering, Cancer Institutes, Department of Oncology, Key Laboratory of Breast Cancer in Shanghai, The Shanghai Key Laboratory of Medical Epigenetics, Shanghai Key Laboratory of Radiation Oncology, The International Co-laboratory of Medical Epigenetics and Metabolism, Ministry of Science and Technology, Shanghai Medical College, Fudan University, Shanghai 200032, China; Fudan University Shanghai Cancer Center & Institutes of Biomedical Sciences, State Key Laboratory of Genetic Engineering, Cancer Institutes, Department of Oncology, Key Laboratory of Breast Cancer in Shanghai, The Shanghai Key Laboratory of Medical Epigenetics, Shanghai Key Laboratory of Radiation Oncology, The International Co-laboratory of Medical Epigenetics and Metabolism, Ministry of Science and Technology, Shanghai Medical College, Fudan University, Shanghai 200032, China; Department of Thyroid, Breast and Vascular Surgery, Xijing Hospital, Fourth Military Medical University, Xi’an 710032, China; Fudan University Shanghai Cancer Center & Institutes of Biomedical Sciences, State Key Laboratory of Genetic Engineering, Cancer Institutes, Department of Oncology, Key Laboratory of Breast Cancer in Shanghai, The Shanghai Key Laboratory of Medical Epigenetics, Shanghai Key Laboratory of Radiation Oncology, The International Co-laboratory of Medical Epigenetics and Metabolism, Ministry of Science and Technology, Shanghai Medical College, Fudan University, Shanghai 200032, China; Fudan University Shanghai Cancer Center & Institutes of Biomedical Sciences, State Key Laboratory of Genetic Engineering, Cancer Institutes, Department of Oncology, Key Laboratory of Breast Cancer in Shanghai, The Shanghai Key Laboratory of Medical Epigenetics, Shanghai Key Laboratory of Radiation Oncology, The International Co-laboratory of Medical Epigenetics and Metabolism, Ministry of Science and Technology, Shanghai Medical College, Fudan University, Shanghai 200032, China; Fudan University Shanghai Cancer Center & Institutes of Biomedical Sciences, State Key Laboratory of Genetic Engineering, Cancer Institutes, Department of Oncology, Key Laboratory of Breast Cancer in Shanghai, The Shanghai Key Laboratory of Medical Epigenetics, Shanghai Key Laboratory of Radiation Oncology, The International Co-laboratory of Medical Epigenetics and Metabolism, Ministry of Science and Technology, Shanghai Medical College, Fudan University, Shanghai 200032, China; Institute of Immunology, CAS Key Laboratory of Innate Immunity and Chronic Disease, School of Basic Medical Sciences, Division of Life Science and Medicine, University of Science and Technology of China, Hefei 230027, China; Department of Pathology, The Cancer Hospital of the University of Chinese Academy of Sciences (Zhejiang Cancer Hospital), Institute of Basic Medicine and Cancer (IBMC), Chinese Academy of Sciences, Hangzhou 310022, China; Department of Breast Surgery, The Cancer Hospital of the University of Chinese Academy of Sciences (Zhejiang Cancer Hospital), Institute of Basic Medicine and Cancer (IBMC), Chinese Academy of Sciences, Hangzhou 310022, China; Department of Breast Medical Oncology, The Cancer Hospital of the University of Chinese Academy of Sciences (Zhejiang Cancer Hospital), Institute of Basic Medicine and Cancer (IBMC), Chinese Academy of Sciences, Hangzhou 310022, China; Fudan University Shanghai Cancer Center & Institutes of Biomedical Sciences, State Key Laboratory of Genetic Engineering, Cancer Institutes, Department of Oncology, Key Laboratory of Breast Cancer in Shanghai, The Shanghai Key Laboratory of Medical Epigenetics, Shanghai Key Laboratory of Radiation Oncology, The International Co-laboratory of Medical Epigenetics and Metabolism, Ministry of Science and Technology, Shanghai Medical College, Fudan University, Shanghai 200032, China; Fudan University Shanghai Cancer Center & Institutes of Biomedical Sciences, State Key Laboratory of Genetic Engineering, Cancer Institutes, Department of Oncology, Key Laboratory of Breast Cancer in Shanghai, The Shanghai Key Laboratory of Medical Epigenetics, Shanghai Key Laboratory of Radiation Oncology, The International Co-laboratory of Medical Epigenetics and Metabolism, Ministry of Science and Technology, Shanghai Medical College, Fudan University, Shanghai 200032, China; Shanghai Institute of Nutrition and Health, University of Chinese Academy of Sciences, Chinese Academy of Sciences, Shanghai 200031, China; Sun Yat-sen University Cancer Center, State Key Laboratory of Oncology in South China, Guangzhou 510060, China; Fudan University Shanghai Cancer Center & Institutes of Biomedical Sciences, State Key Laboratory of Genetic Engineering, Cancer Institutes, Department of Oncology, Key Laboratory of Breast Cancer in Shanghai, The Shanghai Key Laboratory of Medical Epigenetics, Shanghai Key Laboratory of Radiation Oncology, The International Co-laboratory of Medical Epigenetics and Metabolism, Ministry of Science and Technology, Shanghai Medical College, Fudan University, Shanghai 200032, China; Institute of Immunology, CAS Key Laboratory of Innate Immunity and Chronic Disease, School of Basic Medical Sciences, Division of Life Science and Medicine, University of Science and Technology of China, Hefei 230027, China; Department of Breast Surgery, Xiangya Hospital, Changsha 410008, China; Department of Pathology, The Cancer Hospital of the University of Chinese Academy of Sciences (Zhejiang Cancer Hospital), Institute of Basic Medicine and Cancer (IBMC), Chinese Academy of Sciences, Hangzhou 310022, China; Department of Breast Surgery, Tumor Hospital of Harbin Medical University, Harbin 150081, China; Translational Medicine Research and Cooperation Center of Northern China, Heilongjiang Academy of Medical Sciences, Harbin 150081, China; Fudan University Shanghai Cancer Center & Institutes of Biomedical Sciences, State Key Laboratory of Genetic Engineering, Cancer Institutes, Department of Oncology, Key Laboratory of Breast Cancer in Shanghai, The Shanghai Key Laboratory of Medical Epigenetics, Shanghai Key Laboratory of Radiation Oncology, The International Co-laboratory of Medical Epigenetics and Metabolism, Ministry of Science and Technology, Shanghai Medical College, Fudan University, Shanghai 200032, China; Jiangsu Key Lab of Cancer Biomarkers, Prevention and Treatment, Collaborative Innovation Center for Cancer Medicine, Nanjing Medical University, Nanjing 211166, China

**Keywords:** microbiota, ETBF, BFT-1, NOD1, breast cancer stem cell, chemoresistance

## Abstract

Tumor-resident microbiota in breast cancer promotes cancer initiation and malignant progression. However, targeting microbiota to improve the effects of breast cancer therapy has not been investigated in detail. Here, we evaluated the microbiota composition of breast tumors and found that enterotoxigenic *Bacteroides fragilis* (ETBF) was highly enriched in the tumors of patients who did not respond to taxane-based neoadjuvant chemotherapy. ETBF, albeit at low biomass, secreted the toxic protein BFT-1 to promote breast cancer cell stemness and chemoresistance. Mechanistic studies showed that BFT-1 directly bound to NOD1 and stabilized NOD1 protein. NOD1 was highly expressed on ALDH^+^ breast cancer stem cells (BCSCs) and cooperated with GAK to phosphorylate NUMB and promote its lysosomal degradation, thereby activating the NOTCH1-HEY1 signaling pathway to increase BCSCs. NOD1 inhibition and ETBF clearance increase the chemosensitivity of breast cancer by impairing BCSCs.

## Introduction

The critical role of the microbiota in regulating the tumor response to chemotherapy (Geller et al., 2017; Iida et al., 2013; Viaud et al., 2013) and immunotherapy, such as anti-PD-L1 or anti-CTLA-4 treatment (Matson et al., 2018; Routy et al., 2018; Sivan et al., 2015), has been demonstrated in mouse models. Unique microbiota compositions are found in organs previously considered devoid of microbiota, including the lungs, bones, and breasts, and they modulate carcinogenesis in these sites (Nejman et al., 2020). Microbiota differ between the normal breast and breast cancer as well as among different molecular subtypes of breast cancer (Banerjee et al., 2018; Saleh et al., 2021; Urbaniak et al., 2016). Tumor-resident intracellular microbiota facilitates breast cancer metastasis and progression by secreting toxins or regulating the cellular cytoskeleton (Fu et al., 2022; Parida et al., 2021a). For example, the toxin strain of *Clostridium difficile* drives the tumorigenic phenotype of a subset of colorectal cancer patient-derived mucosal slurries by secreting the toxin TcdB in germ-free Apc^Min/+^ mice (Drewes et al., 2022). Microbiota also affect the efficacy of chemotherapy, and the influence of different tumor-resident microbiota on drug efficacy is related to tumor progression or regression. *Fusobacterium nucleatum* modulates chemotherapy resistance by targeting TLR4 and MYD88 innate immune signaling, as well as specific microRNAs to activate autophagy pathways in colorectal cancer (Yu et al., 2017). The efficacy of taxane-based neoadjuvant chemotherapy (TNC), which is one of the main therapeutic strategies in breast cancer, especially for triple-negative breast cancer (TNBC), is limited by the frequent development of resistance (Seruga et al., 2011). However, the potential role of targeting microbiota in breast cancer chemotherapy has not been studied in detail.

Although *Bacteroides fragilis* (*B. fragilis*) accounts for only 0.5% of the normal colonic flora, it is the most frequently isolated anaerobe from clinical specimens (Jasemi et al., 2021). Most *B. fragilis* are commensal organisms. However, some strains of *B. fragilis* are virulent; these are called enterotoxigenic *B. fragilis* (ETBF) and they induce secretory diarrhea and colonic epithelial damage by producing a proteolytic toxin called *B. fragilis* toxin (BFT) (Chung et al., 2018). BFT induces E-cadherin cleavage and actin rearrangement, leading to cell morphological changes and the acquisition of metastatic ability (Saidi et al., 1997; Wu et al., 1998). BFT-triggered activation of the NF-κB and MAPK pathways increases cytokine secretion and promotes immunosuppressive cell infiltration into the colonized niche (Geis et al., 2015; Kim et al., 2002; Thiele Orberg et al., 2017; Wu et al., 2004). ETBF or BFT enhances cancer cell stemness in colorectal cancer and breast cancer (Liu et al., 2020; Parida et al., 2021b). However, the underlying mechanisms and whether there are cellular receptors for BFT that mediate the downstream signaling remain unclear.

Nucleotide binding oligomerization domain containing 1 (NOD1), a member of the cytosolic pattern recognition receptors, plays a vital role in recognizing microbiota products and danger-related molecular patterns (Maisonneuve et al., 2021). NOD1 binds to bacterial peptidoglycan and activates innate immune responses through NF-κB or MAPK signaling, resulting in the secretion of proinflammatory cytokines (Girardin et al., 2001; Inohara et al., 2000). The involvement of NOD1 in cancer progression remains controversial. In a mouse model of colon carcinogenesis, NOD1 deficiency facilitates colon tumor development from colitis, and depleting the microbiota slows tumor progression in mice (Chen et al., 2008). However, recent studies show that NOD1 is involved in tumorigenesis and is negatively correlated with colon cancer patient survival. NOD1 activation increases cancer cell migration, metastasis, and adhesion in colon and liver cancers (Maisonneuve et al., 2021; Nomoto et al., 2022a). Thus, the functions and regulatory mechanisms of NOD1 in tumor progression need to be further elucidated, especially in different cancer types.

Here, we evaluated the distribution of tumor-resident microbiota in breast tumors from patients treated with TNC and found that the presence of ETBF was negatively associated with the chemotherapy response. ETBF promoted breast cancer cell stemness and chemoresistance by secreting BFT-1, which bound to NOD1. Mechanistic studies showed that BFT-1 stabilized NOD1 protein and NOD1 increased the phosphorylation and lysosomal degradation of NUMB by binding to the NUMB-associated kinase cyclin G-associated kinase (GAK), thereby activating the NOTCH1 signaling pathway. The presence of ETBF and NOD1 expression in tumors predicted a poor response to chemotherapy in breast cancer patients. NOD1 inhibition by Nodinitib-1 together with ETBF clearance by metronidazole (MNZ) increased the chemosensitivity of breast cancer by targeting breast cancer stem cells (BCSCs), providing a promising therapeutic rationale to overcome chemoresistance in breast cancer.

## Results

### The presence of ETBF in tumors is positively associated with a poor response to chemotherapy in breast cancer patients

**Figure 1.**
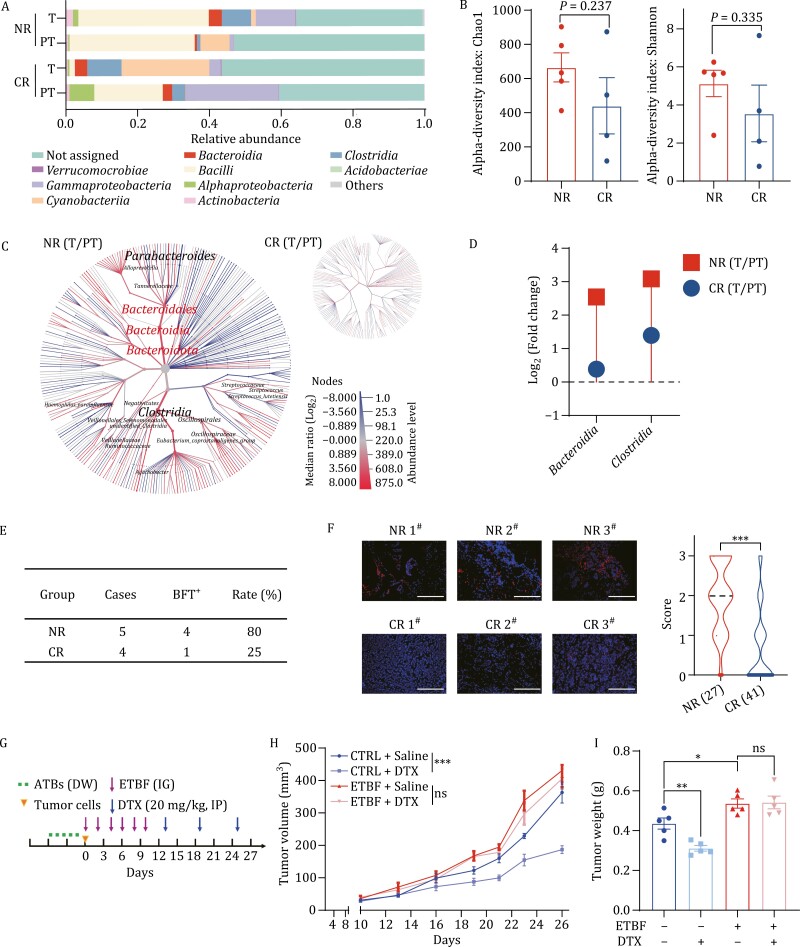
The presence of ETBF in tumors is positively associated with a poor response to chemotherapy in breast cancer patients. (A) The relative abundance of microbiota was assessed by 16S rRNA sequencing at the class level between tumors (T) and para-tumors (PT) from four complete-responders (CR) or five non-responders (NR) to Taxane-based neoadjuvant chemotherapy (TNC). Only the ten most abundant classes were shown. (B) The alpha diversity of tumor-resident microbiota between T and PT from four CR or five NR to TNC was compared by Chao1 and Shannon richness. (C) Heat tree analysis of microbiota communities at species level was assessed by using 16S metabarcoding data between T and PT from NR or CR to TNC. The intensity of the color for each taxa represented the Log_2_ ratio of median proportions of read. Red taxa indicated an enrichment in T and blue taxa was enriched in PT. The names were shown for only taxa with significant differences analyzed using a Wilcox rank-sum test (*P*-value cutoff: 0.05） followed by a Benjamini–Hochberg (FDR) correction for multiple comparison.(D) The relative abundances (fold change) of microbiota communities were assessed in T and PT from CR and NR to TNC. (E) BFT expression was detected by qRT-PCR in T from four CR or five NR to TNC, and the number and the ratio of positive cases were shown. (F) Consecutive slices were stained with fluorescence *in situ* hybridization (FISH) probe against ETBF 16S rRNA to examine the abundance of ETBF in tumors from NR (*n* = 27) and CR (*n* = 41). FISH score was evaluated at a scale of 0–3 based on the percentage of positively stained cells. Representative images (left) and the violin chart of FISH scores (right, mean ± standard error of mean (SEM) were shown. Scale bar: 70 μm, ***P < 0.001. (G) Schematic design for treatment regimen. Balb/c mice were treated with antibiotics (ATBs) via drinking water (DW), and infected with ETBF (1 × 10^9^ colony-forming units (CFU)) by intragastric gavage (IG) every other day for six times. Mice were injected with 3 × 10^4^ 4T1 cells at the fourth mammary fat pads of mice (*n* = 6 for each group). Docetaxel (DTX, 15 mg/kg) were given by intraperitoneal injection (IP) every six-day starting at day 13 (*n* = 6 for each group).(H) The tumor sizes were measured every two days (*n* = 6) and the tumor growth curve was shown. Mice were infected with ETBF (1 × 10^9^ CFU) or water (CTRL) by intragastric gavage (IG) every two days for six times in total (All values were presented as mean ± SEM, ***P < 0.001, vs. CTRL). (I) The tumor weights of 4T1 allograft at the end of experiments were analyzed and graphed. The bar graph was presented as mean ± SEM, *P < 0.05, **P < 0.01, ns: no significance.

To explore the relationship between the microbiota and breast cancer patients’ response to chemotherapy, we first performed 16S rRNA sequencing in tumor (T) and para-tumor (PT) tissues from four complete-responders (CR) and five non-responders (NR) to TNC. Taxonomic profiling at the class or genus level revealed significant differences in the microbiota composition between NR and CR patients (T vs. PT) (Figs. 1Aand S1). The alpha diversity (Chao1 and Shannon) and beta diversity (unweighted distances and weighted distances) of tumor-resident microbiota were higher in NR patients (Figs. 1B and S2), suggesting a potential correlation between tumor-resident microbiota and breast cancer chemoresistance. High-dimensional class comparisons between T and PT from NR or CR were performed using heat tree analysis to determine the critical microbiota responsible for the chemotherapy response. The results showed that *Bacteroidia* and *Clostridia* were significantly enriched in tumors from NR, whereas no significant differences in enrichment were observed in CR (Fig. 1C). The relative abundance (T vs. PT) of *Bacteroidia* and *Clostridia* was further examined in CR and NR patients. *Clostridia* was enriched in tumor tissues of both CR and NR patients, while *Bacteroidia* increased only in NR patients (Fig. 1D), suggesting the potential involvement of *Bacteroidia*, not *Clostridia*, in chemoresistance.

*B. fragilis*, a member of *Bacteroidia*, is a well-studied commensal organism. ETBF, a strain of *B. fragilis,* is associated with tumorigenesis via its oncogenic toxin BFT (Chung et al., 2018; Housseau and Sears, 2010; Parida et al., 2021b; Sears and Pardoll, 2011). To determine whether ETBF was enriched in tumors of NR, we performed qRT-PCR using primers specific to the BFT gene. Nearly 80% tumors from NR were positive for the BFT gene (Fig. 1E), indicating that the presence of BFT-secreting ETBF in tumors may be related to the poor response to chemotherapy. To verify the association between ETBF and the chemotherapy response, we collected 68 tumor biopsies prior to TNC from 27 NR and 41 CR (Table S1), and performed fluorescence *in situ* hybridization (FISH) with a specific probe against ETBF. The fluorescent signals of ETBF were unevenly distributed in breast tumor tissues, and only a small number of cells were stained, indicating a low abundance of ETBF in breast tumor tissue, but almost all tumors of NR were ETBF-positive, and the staining intensity of ETBF in tumors from NR was significantly higher than that in CR tumors (Fig. 1F), indicating that the presence of ETBF in breast tumors is positively associated with a poor response to chemotherapy.

To further demonstrate our conclusions, we injected 4T1 cells into the mammary fat pads of Balb/c mice. These mice were pre-treated with ETBF by intragastric gavage (IG) and subsequently subjected to Docetaxel (DTX) treatment (Fig. 1G). The tumor inhibition rate of DTX-treated was significantly lower in ETBF-infected mice than that in the controls (Fig. 1H and 1I). Overall, these data indicate that the presence of ETBF in tumors is positively associated with a poor response to chemotherapy in breast cancer.

### ETBF presence and NOD1 expression together in breast tumors predict a poor response to chemotherapy

ETBF and its secreted toxic protein BFT regulate cancer cell stemness in colorectal and breast cancers (Liu et al., 2020; Parida et al., 2021b). To explore whether BFT-1 can directly drive resistance to DTX, we administered 4T1 cells injections into the mammary fat pads of Balb/c mice. These mice were pre-treated with BFT-1 via intratumoral injection (IT) and subsequently subjected to DTX treatment (Fig. S3A). The inhibition effect of DTX was significantly smaller in BFT-1-treated allograft tumors than that in the control tumors (Fig. S3B and S3C). Furthermore, BFT-1 increased ALDH^+^ BCSCs in allograft tumors (Fig. S3D), which is similar to that previously seen in ETBF-treated allograft tumors. Overall, these data indicate that the presence of BFT-1 in tumors is positively correlated with a poor response to chemotherapy in breast cancer.

**Figure 2.**
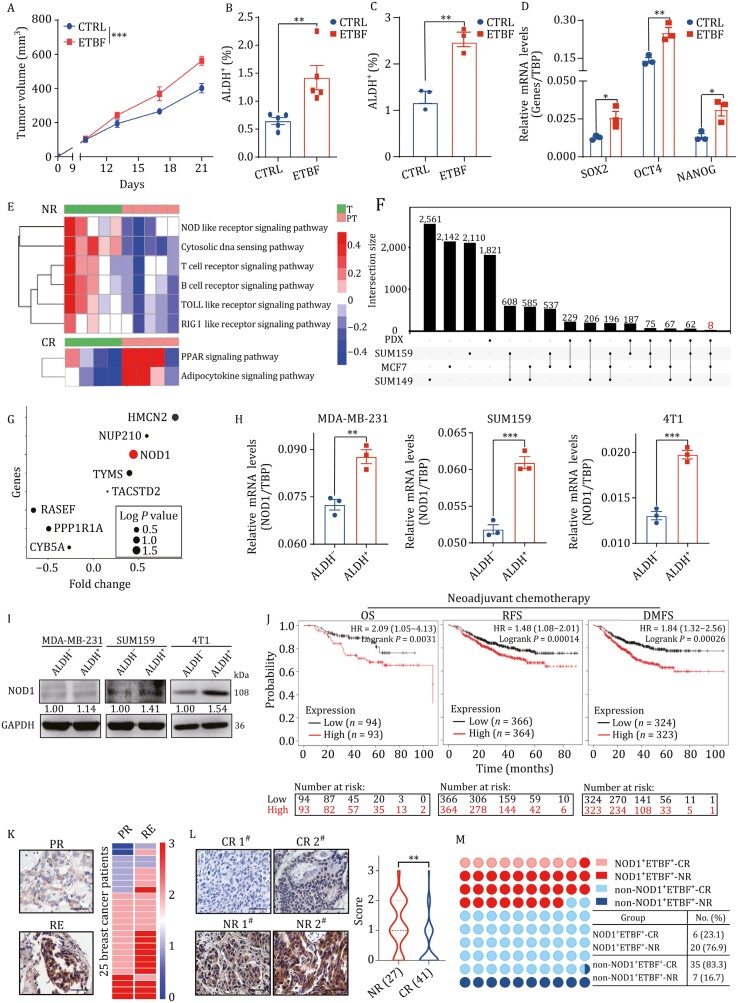
ETBF presence and NOD1 expression together in breast tumors predict a poor response to chemotherapy. (A) The sizes of 4T1 cell allograft tumors treated with or without ETBF were measured every four days in Balb/c mice and the tumor growth curve was shown. Mice were infected with ETBF (1 × 10^9^ CFU) or water (CTRL) by intragastric gavage (IG) every two days for six times in total (All values were presented as mean ± SEM, ***P < 0.001, vs. CTRL). (B) The percentage of ALDH^+^ cells was detected by ALDEFLUOR assay in tumor cells from 4T1 cell allograft tumors treated with or without ETBF. The bar graph was presented as mean ± SEM, **P < 0.01. (C) The percentage of ALDH^+^ cells was detected by ALDEFLUOR assay in SUM159 cells treated with or without ETBF. The bar graph was presented as mean ± SEM of three biological independent experiments, **P < 0.01. (D) The mRNA was isolated from SUM159 cells treated with or without ETBF. The mRNA expression levels of stemness genes SOX2, OCT4 and NANOG were detected by qRT-PCR. The bar graph was presented as mean ± SEM, *P < 0.05, **P < 0.01. (E) Pathway enrichment signatures were assessed by gene set enrichment analysis (GSEA) in T compared to PT from NR and CR to TNC. The rows corresponded to canonical pathways from the Reactome Pathway Database and the columns corresponded to tissue samples. The blue or red color of each cell referred to the − log_10_ (*P*-value). (F) The overlapped stemness-relative genes were analyzed in ALDH^+^ cancer stem cells compared to ALDH^-^ cancer cells from three breast cancer cell lines (SUM159, MCF7, and SUM149) and two breast cancer PDX (overlap gene list) models. Totally, eight overlapped genes were detected in all three cell lines and two PDX models. (G) A bubble chart showed the log*P* value and fold change (FC) at mRNA expression level of above overlapped stemness-relative genes in T from CR compared to T from NR as in (E). (H) The mRNA expression of NOD1 in both ALDH^+^ and ALDH^−^ cells sorted from MDA-MB-231, SUM159 and 4T1 cells was analyzed by qRT-PCR. The bar graph was presented as mean ± SEM of three biological independent experiments, **P < 0.01, ***P < 0.001.(I) NOD1 protein level in both ALDH^+^ and ALDH^−^ cells sorted from MDA-MB-231, SUM159 and 4T1 cells was analyzed by Western blot. (J) The probability of overall survival (OS), relapse free survival (RFS), and distant metastasis-free survival (DMFS) of breast cancer patients received neoadjuvant chemotherapy was analyzed according to NOD1 mRNA expression and the statistical significance was analyzed by log-rank test. Patients were divided into a high-risk group and a low-risk group according to the median value of the NOD1 mRNA expression in tumor tissues. The numbers in the bottom part of the figure are “number at risk”. (K) NOD1 expression was analyzed by IHC staining in paired primary tumors (PR) and recurrent tumors (RE) from the same breast cancer patients. The representative IHC staining images of NOD1 were shown (left, Scale bar: 50 μm). The heatmap (right) showed the IHC staining intensity scores of NOD1 in PR and RE from 25 breast cancer patients. (L) NOD1 expression was analyzed by immunohistochemistry (IHC) staining in breast tumor tissues. The representative IHC staining images of NOD1 were shown (left, Scale bar: 50 μm). Violin plot (right) was shown for the comparison of IHC staining intensity scores of NOD1 between NR (*n* = 27) and CR (*n* = 41) to TNC. The bar graph was presented as mean ± SEM, *P < 0.05.(M) The correlation between NOD1 expression and ETBF presence in tumor tissues from NR or CR to TNC was assessed by IHC staining for NOD1 and FISH staining for ETBF 16S rRNA.

Based on these findings, we speculated that the chemoresistance of NR was due to BCSCs, which might be regulated by ETBF (Fig. 1G–I) and BFT-1 (Fig. S3). To verify this hypothesis, we injected 4T1 cells into the mammary fat pads of Balb/c mice infected with ETBF or NTBF by IG (Fig. S4A). The growth rate and size of allograft tumors were significantly higher in ETBF-infected mice (Fig. 2A), but not NTBF-infected mice (Fig. S4B and S4C). The percentage of ALDH^+^ BCSCs was approximately 2-fold higher in ETBF-infected allograft tumors (Fig. 2B). While the stemness of NTBF-infected allograft tumors (Fig. S4D). In addition, treatment of breast cancer cells with ETBF *in vitro* significantly increased the percentage of BCSCs (Fig. 2C) and upregulated the expression of stemness genes (Fig. 2D). Thus, these results indicated that ETBF increases breast cancer cell stemness *in vivo* and *in vitro*.

To identify the signaling pathways involved in the stemness-promoting effect of ETBF, we performed RNA-seq on the same samples used for 16S rRNA sequencing. In tumors from NR, three signaling pathways related to innate immunity, including NOD-like receptor, TOLL-like receptor, and RIG I-like receptor signaling pathways, were highly enriched according to gene set enrichment analysis (GSEA) (Figs. 2E and S5). We also compared the gene expression profiles of ALDH^+^ BCSCs and bulk cancer cells in three breast cancer cell lines and tumor cells from two patient-derived xenograft (PDX) models. Eight genes were consistently upregulated by >1.5-fold (Fig. 2F), of which NOD1 was the most significantly upregulated gene in tumors of NR (Fig. 2G), suggesting that a potential role of NOD1 in ETBF-increased cancer cell stemness.

NOD1 mRNA levels (Fig. 2H) and protein expression (Fig. 2I) were increased in BCSCs. Clinically high expression of NOD1 was linked to shorter over survival (OS), relapse-free survival (RFS), and distant metastasis-free survival (DMFS) of breast cancer patients who received neoadjuvant chemotherapy (Fig. 2J). Several independent lines of evidence show that NOD1 expression is associated with cancer progression and malignant transformation (Jiang et al., 2020; Zhang, 2022). These studied coupled with our RNAseq data indicated NOD1 might serve as a valuable indicator related to cancer progression and malignant transformation. In addition, we also analyzed NOD1 expression in paired primary tumors (PRs) and recurrent tumors (REs) from the same patient (Table S2) and confirmed the apparent upregulation of NOD1 in recurrent tumors (Fig. 2K). NOD1 expression was considerably higher in pre-TNC tumor tissues from NR than in those from CR, as determined by immunohistochemistry (IHC) (Fig. 2L). These results demonstrate a positive correlation between NOD1 and resistance to TNC in breast cancer. We classified patients according to NOD1 expression and ETBF presence and found that more than 76.9% of NOD1^+^ETBF^+^ patients were resistant to TNC, whereas almost 83.3% of non-NOD1^+^ETBF^+^ patients were sensitive to TNC (Fig. 2M), suggesting that the presence of ETBF and NOD1 expression together in tumors is a biomarker for chemoresistance in breast cancer.

### NOD1 is a functional receptor for BFT-1 and mediates ETBF-enhanced cancer cell stemness

**Figure 3.**
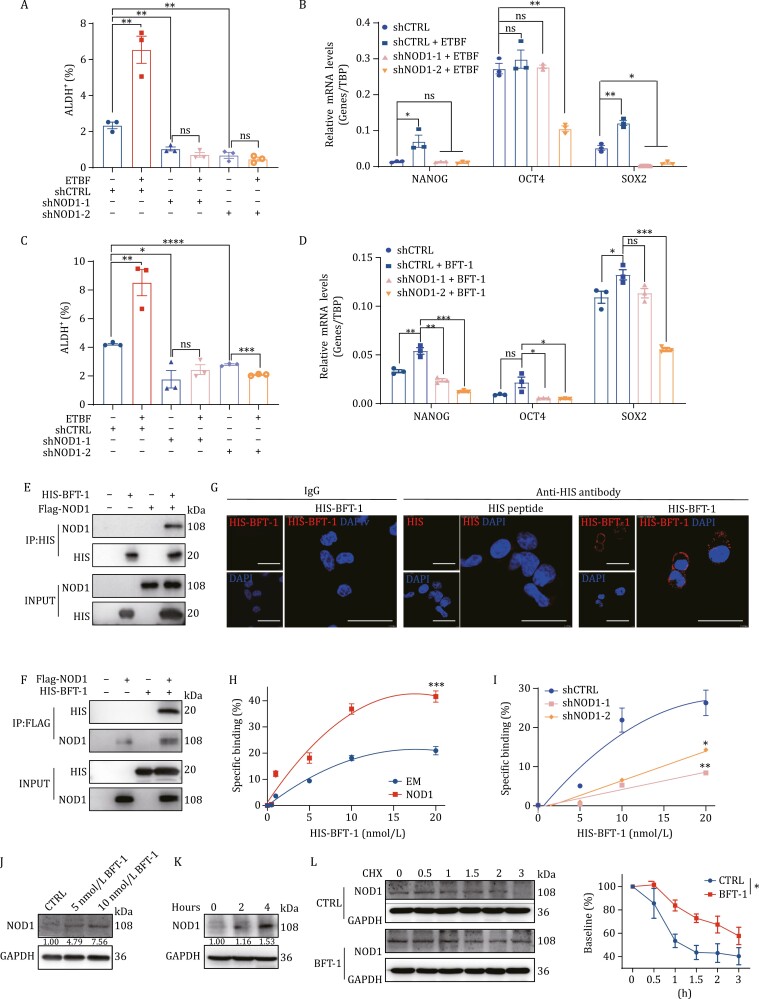
NOD1 is a functional receptor for BFT-1 and mediates ETBF-enhanced cancer cell stemness. (A) The percentage of ALDH^+^ cells was detected by ALDEFLUOR assay in the control (shCTRL) or NOD1-knockdown (shNOD1-1, shNOD1-2) SUM159 cells treated with or without ETBF. The bar graph was presented as mean ± SEM of three biological independent experiments, **P < 0.01. (B) The mRNA was isolated from shCTRL or NOD1-knockdown (shNOD1-1, shNOD1-2) SUM159 cells treated with or without ETBF. The mRNA expression levels of stemness genes SOX2, OCT4, and NANOG were detected by qRT-PCR. The bar graph was presented as mean ± SEM, *P < 0.05, **P < 0.01, ns: no significance. (C) The percentage of ALDH^+^ cells was detected by ALDEFLUOR assay in the control (shCTRL) or NOD1-knockdown (shNOD1-1, shNOD1-2) SUM159 cells treated with or without BFT-1. The bar graph was presented as mean ± SEM of three biological independent experiments, *P < 0.05, **P < 0.01, ****P < 0.0001, ns: no significance.(D) The mRNA was isolated from shCTRL or NOD1-knockdown (shNOD1-1, shNOD1-2) SUM159 cells treated with or without BFT-1. The mRNA expression levels of stemness genes SOX2, OCT4, and NANOG were detected by qRT-PCR. The bar graph was presented as mean ± SEM, *P < 0.05, **P < 0.01, ***P < 0.001, ns: no significance. (E and F) The direct interaction between BFT-1 and NOD1 was assessed by GST pull-down assay. (G) The confocal microscopic images were shown for NOD1-overexpressing SUM159 cells. NOD1-overexpressing SUM159 cells were treated with HIS-peptide or HIS-tagged BFT-1 for two h and stained with anti-HIS antibody. An isotype-matched IgG was used as a control. Cell nuclei were counterstained with DAPI. Scale bar, 20 μm. (H) The empty vector (EM) or NOD1-overexpressing (NOD1) 4T1 cells were treated with or without graded concentrations (0–20 nmol/L) of HIS-tagged BFT-1 for 30 min, and the cells were stained with anti-HIS antibody and the HIS-tagged BFT-1 binding properties were analyzed by flow cytometry. Dead cells were excluded prior to analysis with DAPI staining. All values were presented as mean ± SEM, ***P < 0.001.(I) The shCTRL or NOD1-knockdown (shNOD1-1, shNOD1-2) MDA-MB-231 cells were treated with or without graded concentrations (0–20 nmol/L) of HIS-tagged BFT-1 for 30 min, and the cells were stained with anti-HIS antibody and the HIS-tagged BFT-1 binding properties were analyzed by flow cytometry. Dead cells were excluded prior to analysis with DAPI staining. All values were presented as mean ± SEM, *P < 0.05, **P < 0.01.(J) The protein level of NOD1 in MDA-MB-231 cells treated with BFT-1 at indicated concentrations was analyzed by Western blot. (K) The protein level of NOD1 in MDA-MB-231cells treated with 10 nmol/L BFT-1 for 2 or 4 h was analyzed by Western blot.(L) MDA-MB-231 cells were pretreated with or without 10 nmol/L BFT-1 for 4 h. The culture medium was then replaced with medium containing CHX, and the cells were harvested at the indicated times after CHX treatment. The protein level of was analyzed by Western blot (left) and the NOD1 density was quantified by densitometry using image J (right). Cycloheximide (CHX): 10 μmol/L. All values were presented as mean ± SEM, **P < 0.01, ns: no significance.

The above results indicate that NOD1 might mediate the effect of ETBF on promoting cancer cell stemness. To confirm this hypothesis, human breast cancer cells with NOD1 stable knockdown (Fig. S6C and S6D) were treated with ETBF or BFT-1. The results showed that NOD1-knockdown significantly inhibited ETBF or BFT-1-induced BCSC enrichment (Figs. 3A, 3C, S7B–D and S8A) and suppressed the ETBF or BFT-1-induced upregulation of stemness genes (Figs. 3B, 3D and S8B), suggesting that NOD1 mediated ETBF or BFT-1-enhanced cancer cell stemness. The proportion of BCSCs or stemness gene expression did not change in breast cancer cells treated with non-toxigenic *B. fragilis* (NTBF) (Fig. S9).

ETBF strains are distinguished from NTBF strains primarily by the presence of BFT. BFT-1 is a common toxic protein in ETBF strains isolated from adult feces, which account for approximately 65% of ETBF strains (Jasemi et al., 2021). We hypothesized that BFT-1 secreted by ETBF might interact with NOD1 to exert the biological function of ETBF. To test this, we purified N-terminally FLAG-tagged NOD1 and HIS-tagged BFT-1 proteins. Direct binding between NOD1 and BFT-1 was confirmed using *in vitro* pull-down assays (Fig. 3E and 3F). In NOD1-overexpressing (NOD1) cancer cells (Fig. S6A and S6B) treated with HIS-BFT-1 or HIS peptide, BFT-1 was localized at the plasma membrane as detected by immunofluorescence (Fig. 3G). Evaluation of the ability of BFT-1 to bind to NOD1 using binding assays showed that the specific binding of HIS-BFT-1 was markedly higher in NOD1-overexpressing cancer cells than in empty vector (EM)-expressing cancer cells (Fig. 3H). By contrast, NOD1-knockdown significantly decreased the specific binding effects (Fig. 3I).

Interestingly, we found BFT-1 increased the steady-state level of NOD1 protein in breast cancer cells in a dose- (Fig. 3J) and time- (Fig. 3K) dependent manner. In contrast to the effect on NOD1 protein level, BFT-1 did not significantly alter NOD1 mRNA level (Fig. S10). To validate our conjecture that BFT-1 may increase NOD1 protein level by suppressing its degradation, we treated breast cancer cells with the protein synthesis inhibitor cycloheximide (CHX) and monitored the degradation rate of NOD1 in control cells and BFT-1-pretreated cells, and found that BFT-1 pretreatment significantly attenuated NOD1 degradation (Fig. 3L).

Overall, these data indicate that NOD1, as a functional receptor, is stabilized by BFT-1 and mediates the biological function of BFT-1 secreted by ETBF in breast cancer.

### NOD1 enhances breast cancer cell stemness and chemoresistance

**Figure 4.**
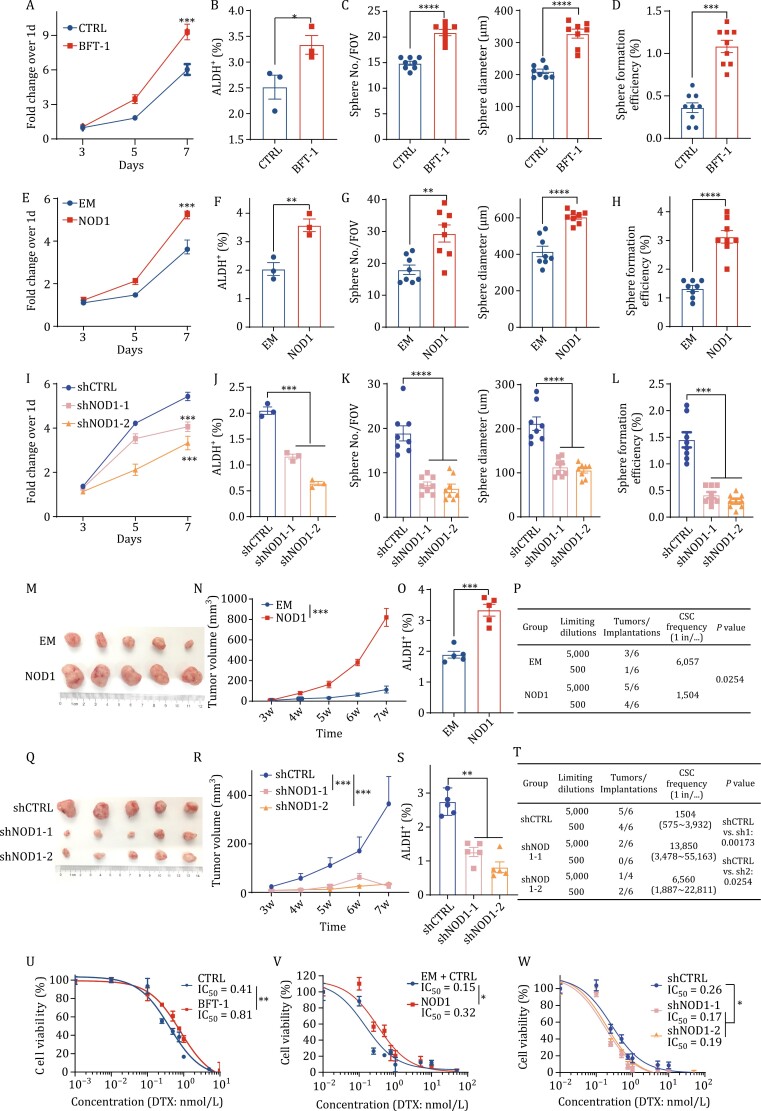
NOD1 positively regulates breast cancer cell stemness and chemoresistance. (A) The cell proliferation was detected by MTT assay in MDA-MB-231 cells treated with or without 10 nmol/L BFT-1. All values between both groups were presented as mean ± SEM, ***P < 0.001. (B) The percentage of ALDH^+^ cells was detected by ALDEFLUOR assay in MDA-MB-231 cells treated with or without 10 nmol/L BFT-1. The bar graph was presented as mean ± SEM of three biological independent experiments, *P < 0.05. (C) The self-renewal ability was determined by primary mammosphere formation assay (sphere number and sphere diameter) in MDA-MB-231 cells treated with or without 10 nmol/L BFT-1. The bar graph was presented as mean ± SEM of three biological independent experiments, ****P < 0.0001. (D) The self-renewal ability was determined by secondary mammosphere formation (sphere formation  efficiency)  assay in MDA-MB-231 cells treated with or without 10 nmol/L BFT-1. The bar graph was presented as mean ± SEM of three biological independent experiments, ***P < 0.001. (E) The cell proliferation was detected by MTT assay in EM or NOD1-overexpressing MDA-MB-231 cells. All values between both groups were presented as mean ± SEM, ***P < 0.001. (F) The percentage of ALDH^+^ cells was detected by ALDEFLUOR assay in EM or NOD1-overexpressing MDA-MB-231 cells. The bar graph was presented as mean ± SEM of three biological independent experiments, **P < 0.01. (G) The self-renewal ability was determined by primary mammosphere formation assay (sphere number and sphere diameter) in EM or NOD1-overexpressing MDA-MB-231 cells. The bar graph was presented as mean ± SEM of three biological independent experiments, ***P < 0.001, ****P < 0.0001. (H) The self-renewal ability was determined by secondary mammosphere formation (sphere formation efficiency) assay in EM or NOD1-overexpressing MDA-MB-231 cells. The bar graph was presented as mean ± SEM of three biological independent experiments, ***P < 0.001. (I) The cell proliferation was detected by MTT assay in shCTRL or NOD1-knockdown (shNOD1-1, shNOD1-2) MDA-MB-231 cells. All values between both groups were presented as mean ± SEM, ***P < 0.001. (J) The percentage of ALDH^+^ cells was detected by ALDEFLUOR assay in shCTRL or NOD1-knockdown (shNOD1-1, shNOD1-2) MDA-MB-231 cells. The bar graph was presented as mean ± SEM of three biological independent experiments, **P < 0.01. (K) The self-renewal ability was determined by primary mammosphere formation assay (sphere number and diameter) in shCTRL or NOD1-knockdown (shNOD1-1, shNOD1-2) MDA-MB-231 cells. The bar graph was presented as mean ± SEM of three biological independent experiments, ***P < 0.001, ****P < 0.0001. (L) The self-renewal ability was determined by secondary mammosphere formation assay (sphere formation efficiency) in shCTRL or NOD1-knockdown (shNOD1-1, shNOD1-2) MDA-MB-231 cells. The bar graph was presented as mean ± SEM of three biological independent experiments, ***P < 0.001. (M and N) 1 × 10^6^ EM or NOD1-overexpressing MDA-MB-231 cells were injected at the fourth mammary fat pads of nude mice (*n* = 5 for each group) and the tumor size was measured once a week. At the end of the experiments, the mice were sacrificed, and the tumor images (M) and the tumor growth curve (N) were shown. All values were presented as mean ± SEM, ***P < 0.001, vs. the EM. (O) The percentage of ALDH^+^ cells was detected by ALDEFLUOR assay in EM or NOD1-overexpressing MDA-MB-231 xenograft tumors. The bar graph was presented as mean ± SEM, ***P < 0.001. (P) The stem cell frequency in EM or NOD1-overexpressing MDA-MB-231 xenograft tumors was calculated by the limited dilution assay. The stem cell frequency and *P* value were calculated based on the positive tumor sites per group. (Q and R) 1 × 10^6^ shCTRL or NOD1-knockdown (shNOD1-1, shNOD1-2) MDA-MB-231 cells were injected at the fourth mammary fat pads of nude mice (*n* = 5 for each group) and the tumor size was measured once a week. At the end of the experiments, the mice were sacrificed, and the tumor images (Q) and the tumor growth curve (R) were shown. All values were presented as mean ± SEM, ***P < 0.001. (S) The percentage of ALDH^+^ cells was detected by ALDEFLUOR assay in tumor cells from shCTRL or NOD1-knockdown (shNOD1-1, shNOD1-2) MDA-MB-231 cell xenograft tumors. The bar graph was presented as mean ± SEM, ***P < 0.001. (T) The stem cell frequency in tumor cells from shCTRL or NOD1-knockdown (shNOD1-1, shNOD1-2) MDA-MB-231 cell xenograft tumors was calculated by the limited dilution assay. The stem cell frequency and *P* value were calculated based on the positive tumor sites per group. (U) Half maximal inhibitory concentration (IC_50_) values of Docetaxel (DTX) were evaluated by MTT assay in MDA-MB-231 cells treated with or without 10 nmol/L BFT-1. All values were presented as mean ± SEM. (V) Half maximal inhibitory concentration (IC_50_) values of Docetaxel (DTX) were evaluated by MTT assay in EM or NOD1-overexpressing MDA-MB-231 cells. All values were presented as mean ± SEM. (W) Half maximal inhibitory concentration (IC_50_) values of Docetaxel (DTX) were evaluated by MTT assay in shCTRL or NOD1-knockdown (shNOD1-1, shNOD1-2) MDA-MB-231 cells. All values were presented as mean ± SEM.

As the only identified virulence factor of ETBF, BFT plays a key role in the progression of cancer (Chung et al., 2018; Liu et al., 2020; Parida et al., 2021b). As expected, BFT-1 significantly increased the proliferation (Figs. 4A and S7A), ALDH^+^ BCSC population (Figs. 4B and S7B) and mammosphere formation ability (Figs. 4C, 4D, S7C and S7D) of breast cancer cells. Based on our above findings that NOD1 was a functional receptor of BFT-1 and was stabilized by BFT-1, we speculated that high NOD1 expression might increase cancer cell stemness and chemoresistance. We next investigated the biological functions of NOD1 in breast cancer cells and found that NOD1-overexpressing in breast cancer cells significantly promoted cancer cell proliferation (Figs. 4E and S11A), increased the ALDH^+^ BCSC population (Figs. 4F and S11B), and upregulated stemness gene expression (Fig. S11E). In addition, NOD1 promoted primary mammosphere formation (including sphere number and diameter) (Figs. 4G and S11C). The secondary mammosphere formation efficiency for the secondary mammosphere was also increased in NOD1-overexpressing cells (Figs. 4H and S11D). These findings suggest that NOD1 enhances breast cancer cell stemness *in vitro*. To provide further evidence, we established shRNA-mediated NOD1-knockdown cell lines. Consistently, the results showed that NOD1-knockdown significantly decreased cell proliferation, mammosphere formation ability, and the BCSC population (Figs. 4I–L and S12A–E).

To verify the results *in vivo*, we injected NOD1-overexpressing cell lines into the fourth mammary glands of five-week-old female mice. NOD1-overexpressing remarkably promoted tumor growth (Figs. 4M, 4N and S11F), and the ALDH^+^ BCSC percentage was elevated by 40% (Figs. 4O and S11G). Consistently, a limited dilution assay (LDA) showed that the frequency of tumor-initiating cells increased by 4-fold in the NOD1-overexpressing group (Fig. 4P), whereas NOD1-knockdown had the opposite effects (Figs. 4Q–T, S12F and S12G).

Previous studies showed that BCSCs contribute to chemoresistance and are enriched after DTX treatment (Awad et al., 2010; Cojoc et al., 2015). We treated breast cancer cells with increasing doses of DTX for 72 h and determined the cytotoxic effects using the MTT assay. Breast cancer cells pretreated with BFT-1 exhibited more resistance to DTX compared to control cells, as indicated by an increase in the half maximal inhibitory concentration (IC_50_) from 0.41 nmol/L to 0.81 nmol/L (Fig. 4U). Similar results were obtained in NOD1-overexpressing breast cancer cells at a higher IC_50_ than that observed in EM cells (Fig. 4V). Conversely, NOD1-knockdown cells were more sensitive to DTX than shCTRL cells (Fig. 4W).

These data indicate that high NOD1 expression either stabilized by BFT-1 or overexpressed enhances cancer cell stemness and chemoresistance in breast cancer.

### NOD1 promotes NUMB lysosomal degradation and subsequently activates the NOTCH1-HEY1 pathway

**Figure 5.**
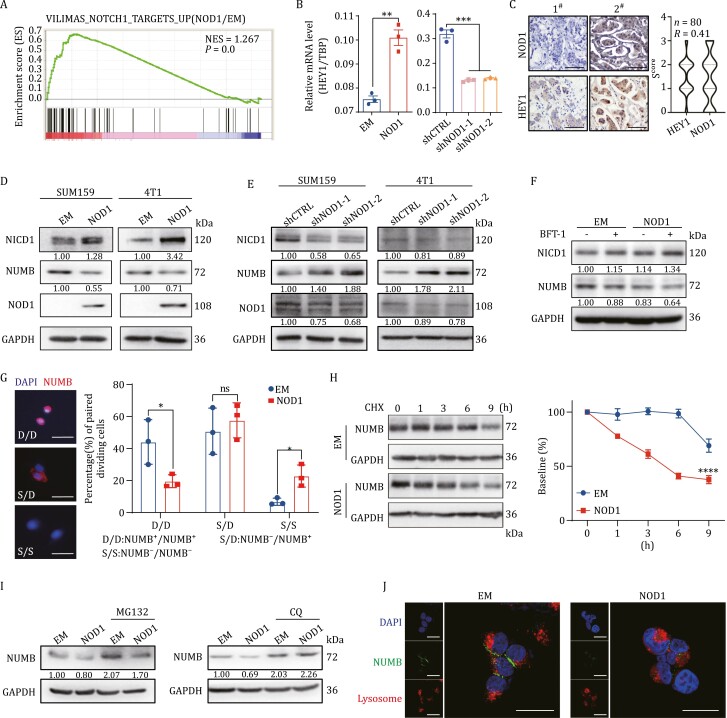
NOD1 promotes NUMB lysosomal degradation and subsequently activates the NOTCH1-HEY1 pathway. (A) Pathway enrichment signatures were assessed by gene set enrichment analysis (GSEA) in EM and NOD1-overexpressing breast cancer cells. The normalized enrichment score (NES) and *P* value were shown in the pictures. (B) The mRNA was isolated from EM or NOD1-overexpressing and shCTRL or NOD1-knockdown (shNOD1-1, shNOD1-2) SUM159 cells. The mRNA expression levels of HEY1 were detected by qRT-PCR. The bar graph was presented as mean ± SEM of three biological independent experiments, **P < 0.01, ***P < 0.001. (C) The correlation between NOD1 expression and HEY1 expression was analyzed in breast tumor tissues by IHC scores using Pearson’s correlation analysis. Representative IHC images of NOD1 and HEY1 in tumor tissues from the same patient (left, scale bar, 100 μm) and violin plot of IHC score of NOD1 or HEY1 (right) were shown (*n* = 80). (D) The protein level of NICD1 and NUMB in EM or NOD1-overexpressing SUM159 or 4T1 cells was analyzed by Western blot. (E) The protein level of NICD1 and NUMB in shCTRL or NOD1-knockdown (shNOD1-1, shNOD1-2) SUM159 or 4T1 cells was analyzed by Western blot. (F) The protein level of NICD1 and NUMB in EM or NOD1-overexpressing SUM159 cells treated with or without 10 nmol/L BFT-1 was analyzed by Western blot. (G) NUMB in two daughter cells was detected by immunofluorescence (IF) staining with NUMB antibody in ALDH^+^ cells sorted from EM or NOD1-overexpressing SUM159 cells. Bar chart showed the frequency of NUMB^−^/NUMB^+^ (S/D), NUMB^+^/NUMB^+^ (D/D), or NUMB^−^/NUMB^−^ (S/S) cell pairs. The bar graph was presented as mean ± SEM of three biological independent experiments (*n* = 50 ALDH^+^ cells/replicate), *P < 0.05, ns: no significance. (H) The degradation rate of NUMB was analyzed by Western blot in EM or NOD1-overexpressing SUM159 cells. The protein level of was analyzed by Western blot (left) and the NUMB density was quantified by densitometry using image J (right). Cycloheximide (CHX): 10 μmol/L. All values were presented as mean ± SEM, ****P < 0.0001. (I) NUMB protein level was analyzed by Western blot in EM or NOD1-overexpressing SUM159 cells treated with or without the lysosomal inhibitor chloroquine (CQ, 1 μmol/L) or the proteasome inhibitor MG132 (1 μmol/L) for 9 h. (J) The localization of NUMB (green) and the lysosomal tracker (red) was analyzed by IF staining in EM or NOD1-overexpressing HEK293T cells treated with CQ for 9 h. Scale bar, 20 μm.

To explore the potential mechanisms underlying the role of high NOD1 expression in promoting breast cancer cell stemness and chemoresistance, we analyzed the RNA-seq data in NOD1-overexpressing breast cancer cells and performed GSEA. As shown in Fig. 5A, NOTCH1 target genes were enriched in NOD1-overexpressing cells. Analysis of the mRNA expression levels of NOTCH1 target genes by qRT-PCR, of which HEY1 expression was the most significantly upregulated by NOD1 (Fig. 5B). In addition, we observed a positive correlation between HEY1 and NOD1 in pre-TNC tumors by IHC analysis (Fig. 5C). These findings indicate that NOD1 might activate the NOTCH1-HEY1 signaling pathway.

NUMB is a tumor suppressor that recruits ubiquitinated E3 ligases to NOTCH receptors, thereby promoting NOTCH1 ubiquitination at the membrane, which induces the degradation of the NOTCH1 intracellular domain (NICD1), thus bypassing its nuclear translocation and downstream activation of NOTCH1 target genes (McGill and McGlade, 2003; McGill et al., 2009). We observed that NOD1-overexpressing significantly inhibited NUMB protein expression and increased NICD1 levels in breast cancer cells (Fig. 5D). By contrast, NOD1-knockdown upregulated NUMB and decreased NICD1 levels (Fig. 5E). Treatment of breast cancer cells with 10 nmol/L BFT-1 significantly downregulated NUMB and increased NICD1 levels in NOD1-overexpressing cells (Fig. 5F). In addition, we knocked down NUMB using lentivirus in NOD-knockdown cells. The NOD1-knockdown-induced phenotype was reversed by NUMB-knockdown (Fig. S13). These results indicate that NOD1 activates the NOTCH1-HEY1 signaling pathway by suppressing NUMB.

NUMB functions as a cell fate determinant for stem cells, including cancer stem cells, and its aberrant expression plays a role in colorectal cancer and glioma stem cells (Abballe et al., 2018). Stem cells maintain the self-renewal ability through asymmetric division, and NUMB segregates into the differentiated cells during this process (Cicalese et al., 2009; Morrison and Kimble, 2006). To determine whether NOD1 increased breast cancer cell stemness by positively regulating BCSC symmetric division, we assessed the pattern of BCSC division *in vitro* by paired-cell analysis and immunostaining of the NUMB protein. BCSCs were sorted and plated as a single cell per well in 96-well plates, and allowed to carry out one division cycle. More than 95% of BCSCs immediately after sorting showed negative NUMB immunostaining at baseline and then underwent predominantly (nearly 60%) asymmetric cell division (Fig. S14). However, 22.8% of BCSCs from NOD1-overexpressing cells produced two NUMB-negative daughter cells after division, whereas 6.7% of BCSCs sorted from EM cells produced two NUMB-negative daughter cells (Fig. 5G), implying that NOD1 promoted the self-renewal of BCSCs.

We examined the mechanisms by which NOD1 downregulated NUMB and observed that the mRNA expression level of NUMB was not affected by NOD1 in breast cancer cells (Fig. S15). In breast cancer cells treated with CHX, the half-life of the NUMB protein was significantly decreased in NOD1-overexpressing cancer cells (Fig. 5H). To identify the degradation pathway responsible for the NOD1-accelerated degradation of NUMB, cells were treated with MG132 and chloroquine (CQ) to block the proteasomal and lysosomal pathways, respectively. As shown in Fig. 5I, MG132 treatment did not reverse NOD1-induced NUMB degradation, although NUMB protein levels were elevated in both EM and NOD1-overexpressing cells.

By contrast, treatment with CQ significantly increased the protein levels of NUMB, and the effect was stronger in NOD1-overexpressing cells than in EM cells (Fig. 5I). This indicated that NOD1-overexpressing might partially promote the lysosomal degradation of NUMB. Furthermore, NUMB was translocated from the cell membrane to the lysosome for degradation in NOD1-overexpressing cells (Fig. 5J). These findings suggest that NOD1 promotes NUMB lysosomal degradation and relieves the inhibition of the NOTCH1 signaling pathway.

### NOD1 interacts with GAK to promote NUMB lysosomal degradation by phosphorylating NUMB

To further explore the mechanisms underlying NOD1-induced NUMB lysosomal degradation, we overexpressed FLAG-tagged NOD1 or EM in HEK293T cells and performed immunoprecipitation using whole cell lysates. Immunoblot analysis of the immunoprecipitated fractions showed that FLAG-tagged NOD1 was enriched in the pull-down assay. By contrast, NOD1 was not detected in the IgG pull-down (Fig. S16A). Co-immunoprecipitated proteins were then analyzed by liquid chromatography–tandem mass spectrometry (LC/MS) analysis. A total of 36 candidate proteins were identified in two duplicates (Fig. S16B), suggesting their potential interaction with NOD1.

**Figure 6.**
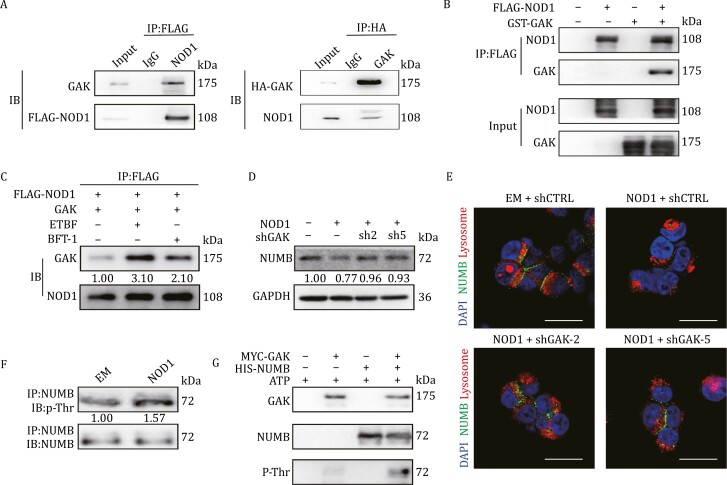
NOD1 interacts with cyclin G-associated kinase (GAK) to promote NUMB lysosomal degradation by phosphorylating NUMB. (A) The direct interaction between NOD1 and GAK was assessed by Co-Immunoprecipitation assay in SUM159 cells. (B) The direct interaction between NOD1 and GAK was assessed by GST pull-down assay. (C) The direct interaction between NOD1 and GAK was assessed by Co-immunoprecipitation assay in NOD1-overexpressing SUM159 cells treated with ETBF or BFT-1. (D) NUMB protein level was analyzed by Western blot in NOD1-overexpressing SUM159 cells with or without GAK-knockdown (shGAK-2, shGAK-5). (E) The localization of NUMB (green) and the lysosomal tracker (red) was analyzed by IF staining in EM or NOD1-overexpressing HEK293T cells with or without GAK-knockdown (shGAK-2, shGAK-5) in the presence of CQ for 9 h. Scale bar, 20 μm. (F) NUMB phosphorylation on Thr residues was analyzed by Western blot in EM or NOD1-overexpressing SUM159 cells. Immunoprecipitation of NUMB in EM or NOD1-overexpressing SUM159 cells was performed and followed by immunoblot analysis for p-Thr. (G) The kinase-substrate interaction between GAK and NUMB protein was analyzed by *in vitro* phosphorylation assay.

We noticed that a member of Numb-associated kinase family, cyclin G-associated kinase (GAK), was among them (Fig. S16C). We speculated whether GAK collaborated with NOD1 to promote NUMB lysosomal degradation. GAK interacted with NOD1, as determined by reciprocal Co-Immunoprecipitation assay (Fig. 6A). The direct interaction between NOD1 and GAK was confirmed using an *in vitro* GST-pull down assay (Fig. 6B). GAK-knockdown in NOD1-overexpressing breast cancer cells (Fig. S17A) significantly inhibited NOD1-induced cell proliferation (Fig. S17B), suppressed BCSC enrichment (Fig. S17C and S17D) and re-sensitized cells to DTX (Fig. S17E), suggesting that GAK-knockdown reversed phenotype induced by NOD1-overexpressing. To determine whether ETBF or BFT-1 influenced the interaction between NOD1 and GAK, we transiently transfected breast cancer cells with FLAG-tagged NOD1 or GAK. At 48 h after transfection, cells were treated with ETBF or 10 nmol/L BFT-1 and immunoprecipitation was performed using an anti-FLAG monoclonal antibody to pull down NOD1. GAK was detected in the anti-FLAG immunoprecipitates from NOD1 co-transfectants, and ETBF or BFT-1 treatment increased the interaction between GAK and NOD1 (Fig. 6C).

To determine whether GAK was involved in the NOD1-induced degradation of NUMB, GAK was knocked down via shRNA in NOD1-overexpressing SUM159 cells, which restored the protein levels of NUMB (Fig. 6D). In addition, GAK-knockdown in the presence of CQ redistributed NUMB to the plasma membrane (Fig. 6E). These results suggest that GAK mediates NOD1-induced NUMB lysosomal degradation. A previous study showed that AAK1, a homolog of GAK, phosphorylated Numb at Thr residues and inhibited its cellular (Sorensen & Conner, 2008). We therefore explored whether GAK regulated NUMB by phosphorylation in NOD1-overexpressing breast cancer cells. We found that the phosphorylation level of Thr residues in NUMB was elevated in NOD1-overexpressing cells (Fig. 6F). An *in vitro* kinase assay showed that GAK phosphorylated the NUMB protein (Fig. 6G). These data suggest that NOD1 recruits GAK to phosphorylate NUMB and promote its degradation.

### NOD1 inhibition and ETBF clearance together increase breast cancer chemosensitivity

The data obtained suggested that ETBF and NOD1 in tumors are potential therapeutic targets to overcome breast cancer chemoresistance. To examine the therapeutic effect of NOD1 inhibition and ETBF clearance in breast cancer, we first treated breast cancer cells with the NOD1 inhibitor Nodinitib-1 alone or in combination with DTX. Nodinitib-1 and DTX effectively suppressed cell proliferation (Fig. S18A), and Nodinitib-1 remarkably reversed DTX-enriched BCSCs in combination treatment (Fig. S18B). Moreover, Nodinitib-1 sensitized breast cancer cells to DTX, as indicated by a decrease in IC_50_ from 0.44 nmol/L to 0.25 nmol/L (Fig. S18C).

**Figure 7.**
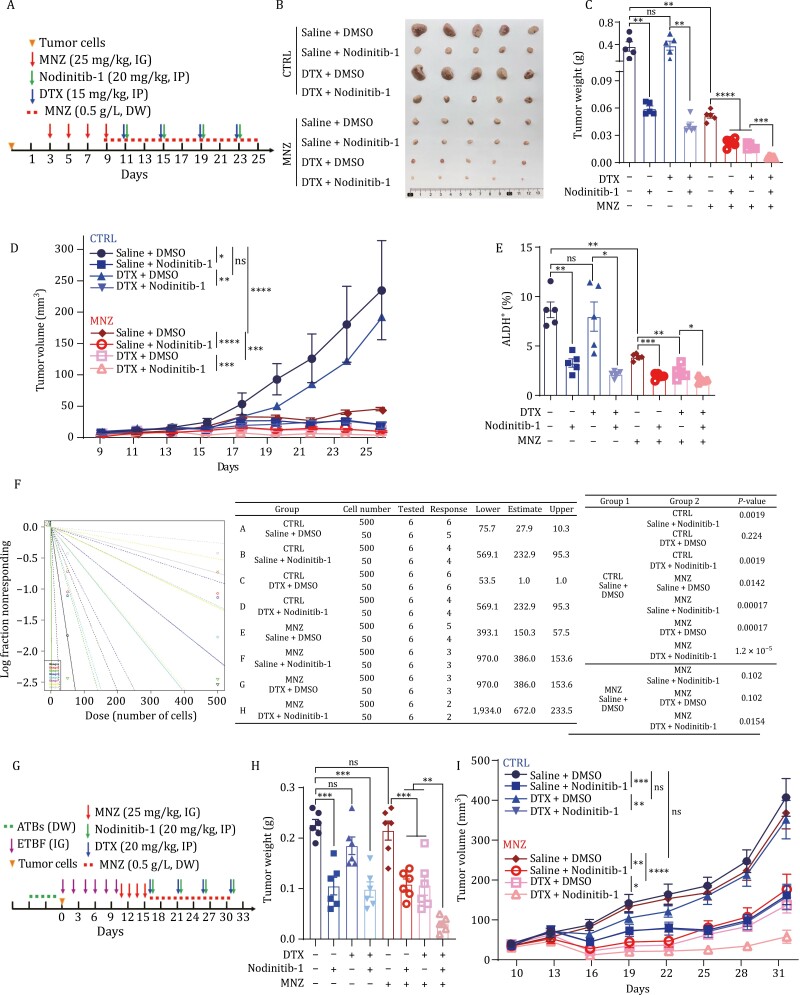
NOD1 inhibition and ETBF clearance together increase breast cancer chemosensitivity. (A) Schematic design for treatment regimen. A 25-day “Trinity” therapeutic strategy was designed for breast cancer cell xenograft tumors. 1000 tumor cells from PDX Lsl17 were injected at the fourth mammary fat pads of Nude mice and were treated with Metronidazole (MNZ, 25 mg/kg) by intragastric gavage (IG) every two days for four times in total and continuously treated with MNZ (0.5 g/L) via drinking water (DW) throughout the experiment. Docetaxel (DTX, 15 mg/kg), Nodinitib-1 (20 mg/kg), or a combination of both drugs were given by intraperitoneal injection (IP) every other day starting at day 11 (*n* = 5 for each group). (B) At the end of experiments, the mice were sacrificed and the tumors were taken out. Representative images of tumors were shown. (C) The tumor weights of PDX Lsl17 tumors at the end of experiments were analyzed and graphed. The bar graph was presented as mean ± SEM, **P < 0.01, ***P < 0.001, ****P < 0.0001, ns no significance. (D) The tumor sizes were measured every two days (*n* = 5) and the tumor growth curve was shown. All values were presented as mean ± SEM, *P < 0.05, **P < 0.01, ***P < 0.001, ****P < 0.0001, ns no significance. (E) The percentage of ALDH^+^ cells was analyzed by ALDEFLUOR assay in cells from PDX Lsl17 tumors subjected to “Trinity” therapeutic strategy. The bar graph was presented as mean ± SEM, *P < 0.05, **P < 0.01, ***P < 0.001, ns no significance. (F) The stem cell frequency was calculated by the limited dilution assay. Tumor cells isolated from PDX Lsl17 tumors subjected to “Trinity” therapeutic strategy were engrafted into mammary fat pads of nude mice at limiting dilution. The stem cell frequency and *P* value were calculated based on the positive tumor sites per group. (G) Schematic design for treatment regimen. 3 × 10^4^ 4T1 cells were injected at the fourth mammary fat pads of Balb/c mice and were treated with ATBs by DW, and infected with ETBF (1 × 10^9^ CFU) by IG for six times. Mice were treated with MNZ (25 mg/kg) for four times by IG and continuously treated with MNZ (0.5 g/L) via DW. Nodinitib-1 (20 mg/kg) alone or combined with DTX (20 mg/kg) were given by IP every five days starting at day 16 (*n* = 6 for each group). (H) The tumor weights of 4T1 allograft tumors at the end of experiments were analyzed. The bar graph was presented as mean ± SEM, ***P < 0.001, ****P < 0.0001, ns no significance. (I) The tumor sizes of 4T1 allograft tumors were measured every three days (*n* = 5) and the tumor growth curve was shown. All values were presented as mean ± SEM, *P < 0.05, **P < 0.01, ***P < 0.001, ****P < 0.0001, ns: no significance.

Most isolates of *B. fragilis* from clinical and colorectal specimens are sensitive to metronidazole (MNZ), and MNZ is the first-line antimicrobial for empirical therapy (Jasemi et al., 2021; Sóki et al., 2020a). In the present study, mice were treated with MNZ at 25 mg/kg by intragastric gavage (IG) once a day. After 4 days, MNZ efficiently eliminated ETBF as measured by fecal colonization (Fig. S19). We then used the NOD1^+^ETBF^+^ TNBC PDX model Lsl17 (Fig. S20A) to evaluate the therapeutic response of the “Trinity” therapeutic strategy with Nodinitib-1, MNZ, and DTX (Fig. 7A). MNZ alone significantly inhibited tumor progression (Fig. 7B–D), and ETBF reinfusion reversed the growth (Fig. S20B and S20C), indicating that clearance of ETBF suppressed the ETBF-dependent breast tumor malignant progression *in vivo*. Nodinitib-1 alone and in combination with DTX significantly inhibited tumor progression (Fig. 7B–D) by decreasing BCSCs (Fig. 7E). The efficacy of the “Trinity” therapeutic strategy on inhibiting tumor growth (Fig. 7B–D) was demonstrated by the decrease in BCSCs, as assessed by the ALDEFLUOR assay and LDA (Fig. 7E and 7F). The therapeutic strategy did not have toxic effects, as shown by HE staining of the livers and spleens (Fig. S20D), and MNZ efficiently eliminated ETBF in tumor tissues (Fig. S20E).

To further explore the potential synergy among DTX, NOD1 inhibition, and ETBF clearance in ETBF-independent breast tumors, we established an ETBF colonized allograft mouse model with 4T1 cells (Fig. 7G). MNZ alone had little effect on tumor progression (Fig. 7H and 7I). However, the ‘Trinity’ therapeutic strategy had a significant inhibitory effect on tumor growth (Fig. 7H and 7I), although other treatments also inhibited tumor growth to some extent (Fig. 7H and 7I). These results suggest that NOD1 plays a crucial role in mediating ETBF-induced malignant progression. Taken together, these results indicate that the combined presence of ETBF and NOD1 expression in tumors contributes to the tumor-malignant phenotype of breast cancer and enrichment of BCSCs. Simultaneous NOD1 inhibition and ETBF clearance are potentially effective chemotherapy strategies.

## Discussion

In this study, we demonstrated that ETBF presence and NOD1 expression contribute to chemoresistance in breast cancer. BFT-1, the virulence factor of ETBF, directly bound to NOD1 and stabilized its protein expression in BCSCs and increased BCSC self-renewal. Investigation of the underlying mechanism showed that NOD1 activated the NOTCH1-HEY1 signaling pathway by enhancing NUMB lysosomal degradation. We showed that the phosphokinase GAK was recruited by NOD1 and phosphorylated NUMB, leading to NUMB translocation from the cell membrane to the lysosome for degradation. NOD1 inhibition and ETBF clearance significantly increased the efficacy of DTX by suppressing BCSCs in breast cancer.

The relevance of gut microbiota in cancer development and treatment response has recently received increased attention (Park et al., 2022). Microbiota influence carcinogenesis in several ways, including microbiota imbalance or ecosystem shifting (Derosa et al., 2021), production of microbiota metabolites (Grajeda-Iglesias et al., 2021), stimulation of pattern-recognition-related signaling (Plovier et al., 2017), and induction of immune responses (Bessell et al., 2020). ETBF promotes tumor formation by producing toxins capable of generating an inflammatory milieu (Chung et al., 2018). However, the relationship between ETBF and breast cancer chemotherapy response as well as the detailed mechanisms underlying ETBF-induced carcinogenesis are unclear. In this study, we showed that ETBF infection significantly promoted breast cancer cell stemness in a NOD1-dependent manner. However, the mammary gland microbiota is complex, and the dysbiosis in cancer may not result from one specific species. In addition to ETBF, other tumor-resident microbiota taxa such as *Clostridia*, *Alphaproteobacteria*, and *Actinobacteria* were enriched in NR. Further studies are required to determine whether a driver microbiota is responsible for carcinogenesis or several microbiotas enriched by dysbiosis coordinately contribute to the process.

Conflicting roles of NOD1 in tumor progression have been reported. NOD1 can promote tumor progression by activating NF-κB signaling in ovarian cancer and esophageal squamous cell carcinoma (Nomoto et al., 2022b; Zhang and Wang, 2022). Intertumoral activation of NOD1 by the gram-negative bacterial peptidoglycan gTri-DAP can enhance the immunosuppressive activity of myeloid cells to foster a tumor-permissive microenvironment in colon cancer (Maisonneuve et al., 2021). Conversely, NOD1 can induce cell apoptosis and inhibit cell proliferation in papillary thyroid carcinoma and hepatocellular carcinoma (Bai et al., 2022; Ma et al., 2020). Most previous studies have focused on NOD1-mediated inflammation and activation of the NF-κB and MAPK signaling pathways. Its trigger factors and physiological functions remain unclear. The initially identified role of NOD1 in sensing peptidoglycan and pathogen infections is well-established (Keestra-Gounder and Tsolis, 2017). In this study, we found that NOD1 was highly expressed in BCSCs and functioned as a receptor for BFT-1, thereby mediating bacterial-induced BCSC enrichment. Elucidating potential new biological functions and molecular mechanisms involving the NOD-like family is essential.

Broad-spectrum antibiotics are commonly used to treat cancer-related infections (Sepich-Poore et al., 2021). However, an increasing number of studies have recently shown that broad-spectrum antibiotics can lead to dysbiosis and negatively impact the efficacy of chemotherapy and immune checkpoint blockade therapies (Chalabi et al., 2020; Hopkins et al., 2020; Pederzoli et al., 2021). Compared with other anaerobic bacteria, *B. fragilis* strains often show cross-resistance to multiple antibiotics (Urbán et al., 2015), whereas almost all clinically isolated *B. fragilis* strains are sensitive to MNZ (Jasemi et al., 2021; Kangaba et al., 2015; Sóki et al., 2020b). MNZ is a spectrum antibiotic, and thus could massively disrupt gut microbiota and kill the commensal microbiome in tumors that are necessary for chemotherapy. Therefore, compounds targeting BFT instead of ETBF might be a promising strategy to promote favorable responses in ETBF^+^ breast cancer patients.

In summary, *in vitro* studies supported by clinical analysis and mouse models showed that the tumor-resident microbiota ETBF mediated chemoresistance in breast cancer by enriching BCSCs. Based on bioinformatics and clinical research, we showed that BFT-1 secreted by ETBF bound to its functional receptor NOD1 and stabilized its protein expression, and then NOD1 interacted with GAK to promote NUMB lysosomal degradation, thus activating the NOTCH1-HEY1 signaling pathway. These findings highlight a new molecular mechanism underlying NOD1-driven BCSC enrichment and suggest that profiling microbiota in the cancer niche is important. In addition, the data provide new clues for refining treatment and improving the chemotherapy response in breast cancer.

## Materials and methods

### Ethics statement

The animal experiments were conducted in accordance with the Guide for the Care and Use of Laboratory Animal of Fudan University and approved by the Fudan University Shanghai Cancer Center Institutional Review Board (JS-113). Informed consent was obtained from all patients before collecting serum and tissue samples. This study was approved by the Fudan University Shanghai Cancer Center Institutional Review Board (050432-4-1212B), the Care and Use of Laboratory Animal of Fudan University (202401FD0003, 202401FS0002) and Harbin Medical University Cancer Hospital Institutional Review Board (IRB: KY2019-08).

### Patients and clinical specimens

Human breast para-tumor and tumor specimens were obtained under Institutional Review Board-approved protocols. All fresh tissues and paraffin sections of para-tumor and tumors from breast cancer patients used in this study were provided by Harbin Medical University Cancer Hospital.

### Reagents

DTX was purchased from Jiangsu Hengrui Medicine Company (H20020543, Jiangsu, China) and diluted to the desired concentrations freshly before each experiment. Concentrations and durations of treatment were indicated in the corresponding legends. The commercial NOD1 inhibitor Nodinitib-1 (HY-18639, MedChemExpress) was used at 5–10 μmol/L for 48–72 h *in vitro* and 20 mg/kg *in vivo*. Metronidazole (MNZ) was purchased from Sigma–Aldrich (M3761). All antibiotics (Ampicillin, Streptomycin, Vancomycin, and Colistin) were purchased from Sangon Biotech (A100339, A100382, A100990, A610318). Lysosome tracker^TM^ Deep Red was purchased from Thermo Fisher Scientific, Inc. (L12492). The HIS-BFT-1 and FLAG-NOD1 protein were custom-designed from Zoonbio Biotechnology (Nanjing, China). Human Recombinant Protein GAK (NM_005255) and NUMB (NM_001005743) were purchased from ORIGENG.

### Cell culture

Human breast cancer cell lines SUM159 from Asterland Bioscience were cultured in Han’s F12 medium (21700-075, Gibco) supplemented with 5% fetal bovine serum FBS (VS500T, Ausbian), 1% streptomycin/penicillin (C0222, Beyotime), 5 mg/mL insulin (BS901, Biosharp) and 1 μg/mL hydrocortisone (H110523, aladdin). MDA-MB-231 and 4T1 were purchased from ATCC and were cultured in RPMI1640 medium (31800-022, Gibco) supplemented with 5% FBS, 4 μg/mL gentamycin, and 1% streptomycin/penicillin. Cell lines were maintained at 37°C in a 5% CO_2_-humidified atmosphere. All cell lines were tested for negative mycoplasma contamination.

### Bacterial strains

Enterotoxigenic *B. fragilis* (ETBF, ATCC 43860) and nontoxigenic *B. fragilis* (NTBF, ATCC 25285) were kindly gifted by Prof. Jing-Yuan Fang (Renji Hospital, School of Medicine, Shanghai Jiao Tong University, Shanghai, China). ETBF and NTBF were cultured in brain heart infusion broth (CM1135, OXOID) supplemented with Yeast Extract (LP0021B, OXOID), K_2_HPO_4_ (A100705-0500, Sangon), Resazurin (R7017, Sigma), l-cysteine (C1276, Sigma), Hemin (51280, Sigma), and Vitamin K1 (A606528, Sangon Biotech) at 37°C under anaerobic condition (DG250, Don Whitley Scientific, West Yorkshire, UK).

### Plasmid construction, lentivirus production, and transfection

Human NOD1 full-length ORF was amplified using cDNA of SUM159 cell line and cloned into the pSIN-EF1a-IRES-puro vector using the OneStep kit (#C112, Vazyme). FLAG tag was introduced at the C-terminus of NOD1. Human GAK Full-length ORF with HA tag was amplified using cDNA of SUM159 cell line and cloned into the pLVX-Puro vector. cDNA of BFT-1 was synthesized at Sangon Biotech (Shanghai, China) and cloned into the pGEX-6p-1 vector. FLAG-tagged NOD1, HA-tagged NUMB, and HIS-tagged GAK were subcloned into the pGEX-6p-1 vector for GST pull-down assay. The shRNA sequence of NOD1 and GAK were cloned into pLKO.1-puro vector (#8453, Addgene, USA). Plasmid DNA was transduced into 293T cells to generate high titers of lentivirus. Breast cancer cells were then transfected with lentivirus to establish stable cell lines. Bacterial expression plasmids were transduced into the BL21(DE3) pLysS strain to produce the desired protein. The primers used for cloning are listed in Table S3.

### Total RNA isolation and qRT-PCR

Total RNA was extracted from cells or tissues using RNAiso Plus (Takara Bio), and complementary DNA (cDNA) was obtained from 1 μg RNA using HiScript II 1st Strand cDNA Synthesis kit (Vazyme Biotech). Quantitative real-time PCR (qRT-PCR) was performed using the AceQ Universal SYBR qPCR Master Mix (Q511, Vazyme), and signals were collected using the 7300Plus Real-Time PCR System (Applied Biosystems). Primers used for qRT-PCR are listed in Table S4.

### RNA-seq and data processing

Differential gene analysis was performed by RNA-seq in tumor and para-tumor of 9 patients and NOD1-overexpressing cell lines. After RNA was extracted with RNAiso Plus, the RNA integrity (RNA Quality Number) was verified by Bioptic Qsep100. RNA-seq library was prepared using NEB Next Ultra Directional RNA Library Prep Kit for Illumina (New England Biolabs, Beverly, MA, USA). Sequencing was performed on the HiSeq3000 platform (Illumina, San Diego, CA, USA). Differential genes by RNA-seq were used for GSEA pathway enrichment, and *P* values less than 0.05 were considered statistically significant.

### Breast cancer cell isolation, flow cytometry, and sorting

Patient-derived Xenograft (PDX) was constructed from TNBC patient tumor sample and has been stably passaged for two generations in NOD/SCID mice. The tumor subtype was verified by IHC staining for ER, PR, and HER2. After the mice were sacrificed, xenograft or PDX tumors harvested from the mice were cut into small pieces and resuspended using a collagenase-hyaluronidase digestion solution (STEMCELL Technologies, USA). Tumors were digested with a shaker at 37°C for 1 h. The cell suspension was filtered with a 40 μm filter, and aggregates were removed. Red blood cells were lysed using ammonium chloride solution (#07850, STEMCELL Technologies, USA) for 5 min at room temperature. The cell suspension was centrifuged at 1,200 rpm for 5 min and resuspended for subsequent experiments. Fluorescence-conjugated antibody staining or ALDEFLUOR assay was performed using 0.5–1 million cells. For HIS-tag staining, dissociated single cell suspensions were treated with anti-HIS antibody as the primary antibody (diluted 1:50, isotype IgG as negative control) and PE-conjugated donkey anti-rabbit IgG (Jackson ImmunoResearch) as the secondary antibody (diluted 1:200). For the ALDEFLUOR assay, dissociated single cells were resuspended in ALDEFLUOR buffer containing the substrate BAAA, DEAB was added as a negative control, and incubated at 37°C for 40 min. After staining, the detection was performed in 1× PBS containing 2% FBS and DAPI (D9564, Sigma). Flow cytometry analysis and cell sorting were performed with Moflo Astrios or CytoFlex (Beckman Coulter) and analyzed by Summit 6.3 software.

### Immunofluorescence and confocal imaging

5 × 10^4^ cells were seeded in a chamber (154526, Thermo Scientific, USA), cultured in a 5% CO_2_ 37°C cell incubator for two days, then fixed with cold methanol and treated with 0.15% Triton X100 (TB0198, Sangon Biotech). Animal non-immunized serum (SP KIT-B, Maxvision) was used for blocking. Samples were incubated with primary antibody at 4°C overnight and then incubated with secondary antibody at room temperature for 30 min. Nuclei were stained with DAPI (Invitrogen). Images were captured by confocal microscopy (Leica TCS SP5) with a 63× oil objective.

### MTT and DTX chemosensitivity assay

Breast cancer cells were seeded on 96-well culture plates, and after cell adhesion, a medium with gradient concentrations of DTX was added. Cells were incubated with or without the drug for 72 h. MTT (Biosharp Life Science) was added to the wells (final concentration: 0.5 mg/mL), and the plates were incubated for 4 h at 37°C. The supernatant was removed, and MTT formazan crystals were then resolubilized by adding 100 μL of DMSO. The optical density value was measured at 490 nm after dissolution.

### Mammosphere formation assay and spheroid digestion

Breast cancer cells were plated in 96-well ultra-low attachment plates (#3471, Corning, USA) with MammoCult Human Medium Kit (#05620, STEMCELL Technologies, USA) supplemented with 4 μg/mL heparin (#07980, STEMCELL Technologies, USA), 1 μg/mL hydrocortisone (Sigma, St. Louis, USA). After incubation, the spheroids were collected and trypsinized with 0.25% trypsin at 37°C and centrifuged at 1,200 rpm for 5 min at 4°C. Single-cell suspensions were used for subsequent experiments.

### Western blot

Protein lysis of cells or tissues was extracted with RIPA buffer (Beyotime Biotechnology) and quantified with a BCA kit (Thermo Fisher). The protein lysate and loading buffer were mixed proportionally and denatured for 10 min. Protein samples were separated by SDS-PAGE and transferred to PVDF membranes (Millipore). 5% bovine serum albumin in TBS in 0.1% Tween-20 (TBST) was used for blocking and antibody dilution. The primary antibody was incubated overnight at 4°C and then with HRP-conjugated secondary antibodies for 30 min at room temperature. Chemiluminescence was detected using an ImageQuant LAS 4000 Micro Imaging System (GE). Antibody information used for western blot is listed in Table S5.

### IHC staining

Tumors or organ tissues were fixed with formalin solution for 24 h, dehydrated with graded alcohol, and then embedded in paraffin. For IHC staining, sectioned samples were deparaffinized three times in xylene and then hydrated. Endogenous peroxidase was inactivated by 3% hydrogen peroxide in methanol, and the antigens were thermally repaired with citric acid under high temperature and high pressure. Animal non-immune serum (SP KIT-B, Maxvision) was used for blocking. Sections were incubated with the primary antibody overnight at 4°C. The next day, sections were washed three times with PBS, incubated with secondary antibody at room temperature for 20 min, and stained with a DAB detection kit (DAB-0031, MaxVision). Nuclei were stained with hematoxylin (ZLI-9610, ZSGB-BIO). Sections were dehydrated with graded alcohol and xylene and sealed with neutral resin (BL085A-100g, Biomiky). Slice species were scored using both staining intensity and percentage of stained cells. The intensity was graded as 3 for solid staining, 2 for moderate staining, 1 for weak staining, and 0 for negative staining. The following antibodies and dilutions were used for IHC: NOD1 (1:50, ab189435, Abcam), HEY1 (1:50, 19929-1-AP, ProteinTech), peroxidase-conjugated goat anti-mouse/rabbit (KIT -5010, MaxVision).

### Immunoprecipitation and mass spectrometry

Cells were lysed with EBC buffer (50 mmol/L Tris, 120 mmol/L NaCl, 0.5% NP40, pH 7.5) to obtain a protein lysate. Magnetic FLAG-Beads (Sigma-Aldrich) or anti-HIS antibody-coupled protein A/G agarose Beads (Thermo Fisher) were added to the protein lysate, and samples were rotated overnight at 4°C. After washing with NETN buffer (20 mmol/L Tris, 100 mmol/L NaCl, 0.5% NP40, 1 mmol/L EDTA, pH 8.0), the complexes were eluted and subjected to Western blot. For mass spectrometry analysis, cells were pretreated with MG132 (10 μmol/L, 2 h) before being harvested. Samples were separated by SDS-PAGE electrophoresis, dissolved with 50 mmol/L ammonium bicarbonate solution, and trypsinized at 37°C for 20 h. After centrifugation, the supernatant was lyophilized, desalted, and vortexed to dissolve by adding 0.1% formic acid. The samples were then centrifuged, and the supernatant was added to vials for detection by mass spectrometry (Orbitrap Elite). The search database was Maxquant. Antibody information used for Western blot and Immunoprecipitation is listed in Table S5.

### *In vitro* kinase assay

GST fusion proteins (GAK and NUMB) were purified from the BL21(DE3) pLysS strain and added to the ATP-based kinase buffer system (50 nmol/L HEPES, 100 mmol/L NaCl, 10 mmol/L MgSO_4_, 2 mmol/L DTT, 4 mmol/L EDTA, pH 7.2) (final concentration: 50 μmol/L) The reaction was conducted at 30°C for 30 min, and then mixed with loading buffer for protein denaturation and subsequent Western blot.

### *In vivo* tumorigenicity

Four or five-week-old female Balb/c mice or Nude mice were purchased from Vitalriver (Beijing, China) and housed in standard animal cages under a Specific-pathogen-free facility at 23–25°C on a 12-h light/dark cycle in the Department of Laboratory Animal Science of Fudan University. The animal experiments were conducted in accordance with the Guide for the Care and Use of Laboratory Animal of Fudan University and approved by the Fudan University Shanghai Cancer Center Institutional Review Board (JS-113). Mice were orthotopically injected at the fourth pair of mammary fat pads with breast cancer cells.

### Fluorescence *in situ* hybridization (FISH)

Sections were deparaffinized in three changes of xylene at RT for 10 min each and then were dehydrated in two changes of 100% EtOH at RT for 5 min each. After air-dried, slides were incubated in 0.2 mol/L HCl at RT for 20 min and then rinsed by ddH_2_O and 2× SSC. Prewarmed 1 mol/L NaSCN solution was used to treat slides for 10 min at 80°C. After washing by 2× SSC, slides were incubated in pre-warmed 0.01 mol/L HCl for 10 min.

DNA-FISH probe was designed based on sequences of ETBF, and probe EUB 338 was used to visualize the entire bacterial population in the specimens. Approximately 10 μl of the probe was applied to the target area on the slide and covered with a cover slip. Slides were co-denatured and probed for 5 min at 90°C and then incubated in a humidified environment at 37°C overnight. After washing by 2× SSC/0.1% Tween 20 at 45°C for two times and 0.5× SSC/0.1% Tween 20 at 45°C for two times, DAPI/Antifade solution was applied to the hybridized area. Images were captured with a 20× objective.

### Detection of total bacteria and ETBF in feces

Genomic DNA was extracted from equal-weight mouse stool samples using the SPINeasy DNA Kit for feces (116531050, MPbio). The total bacteria and ETBF contents were determined by qPCR for each sample. Each reaction contained 100 ng of DNA and was assayed in triplicate.

### 16S rRNA sequencing and data analysis

Total genomic DNA was extracted from the samples using CTAB/SDS method, and DNA concentration and purity were checked by 1% agarose gel. Different regions of the 16S rRNA/ITS gene (16S V4, 16S V3, 16S V3-V4, 16S V4-V5, and ITS) were amplified using specific primers. 15 μL of Phusion High-Fidelity PCR Master Mix (New England Biolabs) was used for each PCR reaction: 0.2 μmol/L of forward and reverse primers and about 10 ng template DNA. Thermal cycling consisted of initial denaturation at 98°C for 1 min, followed by 30 cycles of denaturation at 98°C for 10 s, annealing at 50°C for 30 s, and elongation at 72°C for 30 s. Finally, 72°C for 5 min. Mix the same volume of I× loading buffer (contained SYB green) with PCR products and operate electrophoresis on 2% agarose gel for detection. PCR products were purified using Qiagen Gel Extraction Kit (Qiagen, Germany). Sequencing libraries were generated using the TruSeq DNA PCR-Free Sample Preparation Kit (Illumina, USA) according to the manufacturer’s recommendations, with index codes added. Library quality was assessed on a Qubit@2.0 fluorometer (Thermo Scientific) and an Agilent Bioanalyzer 2100 system. Finally, the library was sequenced on the Illumina NovaSeq platform. Sequencing data were merged with paired-end reads using FLASH (VI.2.7) and QIIME (V1.9.1) quality control process for data filtering and finally using the UCHIME algorithm to compare the tags with a reference database (Silva database) (UCHIME algorithm) to detect chimeric sequences, and then remove chimeric sequences to get valid Tags.

### Statistical analysis

All data are expressed as mean ± SEM and were analyzed using GraphPad Prism 6 software. Unless otherwise stated, comparisons between the two data groups were performed with an unpaired Student’s two-way *t*-test. Analysis of variance (ANOVA) was used for multiple comparisons. A two-sided log-rank (Mentel-Cox) test was used to evaluate survival curve analysis. *P* < 0.05 was considered statistically significant, and *P* values in the figure are indicated with the following asterisks, *P < 0.05, **P < 0.01, ***P < 0.001, ****P < 0.0001, ns, no significance.

## Supplementary Material

pwae005_suppl_Supplementary_Tables_S1-S5_Figures_S1-S20

## Data Availability

RNA-seq and 16S rRNA data profiles from this study have been deposited in the NCBI under accession code PRJNA1077768. All other data supporting the results can be found in this paper and its Supplementary Materials. All other relevant data can be obtained from the corresponding authors upon request.

## Code availability

All the custom codes are available from the corresponding authors upon reasonable request.
