# Supplementary material for: Microbiota enterotoxigenic Bacteroides fragilis-secreted BFT-1 promotes breast cancer cell stemness and chemoresistance through its functional receptor NOD1
Source: Protein Cell. 2024 Mar 4;15(6):419–40. doi: 10.1093/procel/pwae005 (PMC11131025; doi:10.1093/procel/pwae005)
Supplement: pwae005_suppl_Supplementary_Tables_S1-S5_Figures_S1-S20 [file pwae005_suppl_supplementary_tables_s1-s5_figures_s1-s20.pdf]

Supplementary Materials

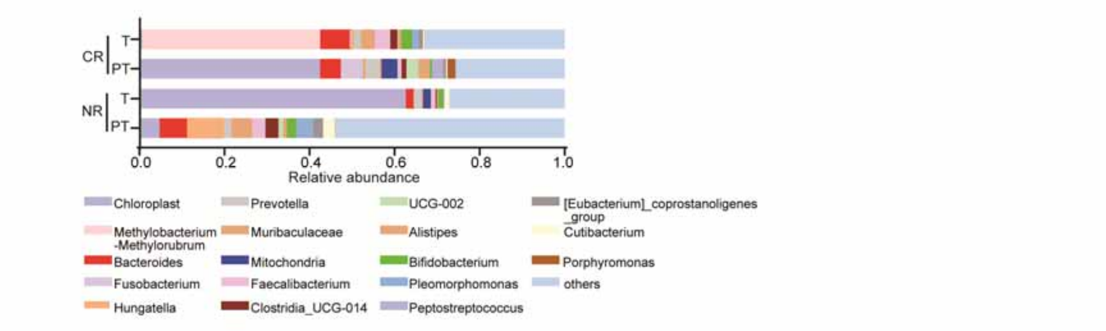

Figure. S1 The relative abundance of microbiota is assessed by 16S rRNA sequencing at the genus level.

The relative abundance of microbiota was assessed by 16S rRNA sequencing at the genus level between T and PT from four CR or five NR to TNC. Only the 18 most abundant genus were shown.

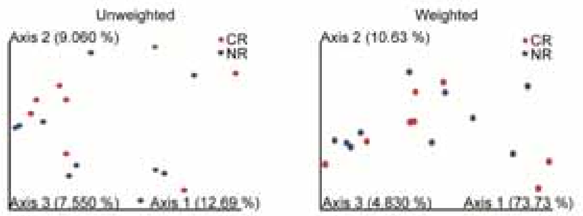

Figure. S2 The beta diversity analysis of T and PT from four CR or five NR patients to TNC.

PCoA 2D plots of β-diversity analysis of T and PT from four CR or five NR patients to TNC. Between-sample dissimilarities were measured by unweighted distances (*p*=0.479) and weighted distances (*p*=0.759).

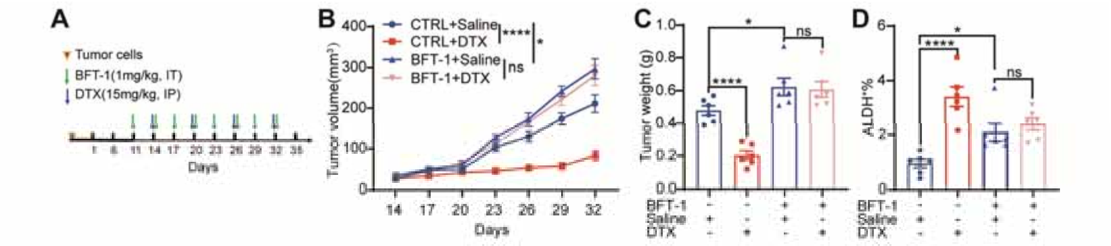

Figure. S3 BFT-1 induces DTX resistance and stemness in vivo.

(A) Schematic design for treatment regimen. Balb/c mice were injected with $3x10^4$ 4T1 cells at the fourth mammary fat pads of mice (n=6 for each group). Mice were treated with BFT-1 (1mg/kg) or PBS (CTRL) by intratumoral injection (IT) every two days for eight times in total. Docetaxel (DTX, 15 mg/kg) was given by intraperitoneal injection (IP) every other day starting at day 14 (n=6 for each group).

(B) The tumor sizes of 4T1 cell allograft were measured every three days (n=6) and the tumor growth curve was shown (All values were presented as mean ± SEM, * p< 0.05, *** p< 0.001, ns no significance).

(C) The tumor weights of 4T1 cell allograft at the end of experiments were analyzed and graphed. The bar graph was presented as mean ± SEM, * p< 0.05, **** p< 0.0001, ns no significance.

(D) The percentage of $ALDH^+$ BCSCs was detected by ALDEFLUOR assay in tumor cells at the end of experiments. The bar graph was presented as mean ± SEM, * p< 0.05, **** p< 0.0001, ns no significance.

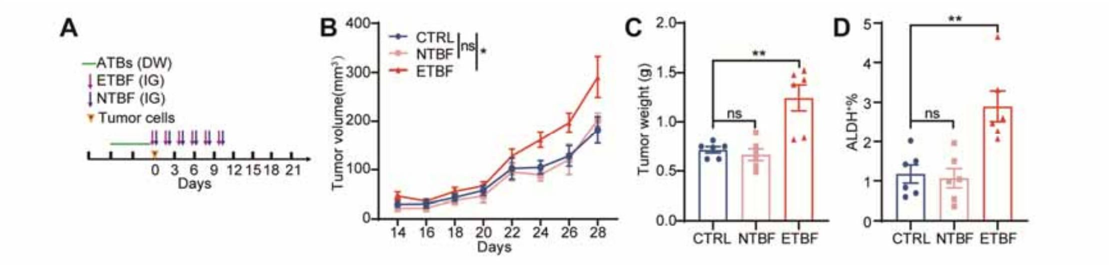

Figure. S4 ETBF promotes the tumor growth of 4T1 cells in Balb/c mice.

(A) Schematic design for treatment regimen. Balb/c mice were treated with ATBs via DW, and infected with ETBF (1x10^9 CFU) or NTBF (1x10^9 CFU) by IG every other day for six times. Mice were injected with 3x10^4 4T1 cells at the fourth mammary fat pads of mice (n=6 for each group).

(B) The sizes of 4T1 cell allograft tumors treated with ETBF or NTBF were measured every other day in Balb/c mice and the tumor growth curve was shown. Mice were infected with ETBF (1x10^9 CFU), NTBF (1x10^9 CFU) or water (CTRL) by intragastric gavage (IG) every two days for six times in total (All values were presented as mean ± SEM, * $p< 0.05$, ns no significance vs CTRL).

(C) The weights of 4T1 allograft tumors in Balb/c mice treated with ETBF or NTBF were shown. The bar graph was presented as mean ± SEM, ** $p< 0.01$, ns no significance.

(D) The percentage of ALDH$^+$ cells were detected by ALDEFLUOR assay in tumor cells from 4T1 cell allograft tumors treated with ETBF or NTBF. The bar graph was presented as mean ± SEM, ** $p< 0.01$, ns no significance.

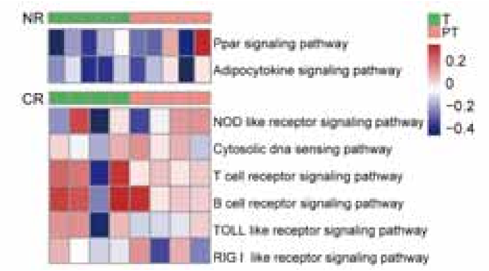

Figure. S5 Pathway enrichment signatures are assessed by gene set enrichment analysis (GSEA) in T compared to PT.

Pathway enrichment signatures were assessed by gene set enrichment analysis (GSEA) in T compared to PT from both NR and CR to TNC. The rows stated to canonical pathways from the Reactome Pathway Database and the columns stated to tissue samples. The blue or red color of each cell referred to the -log10 (p-value).

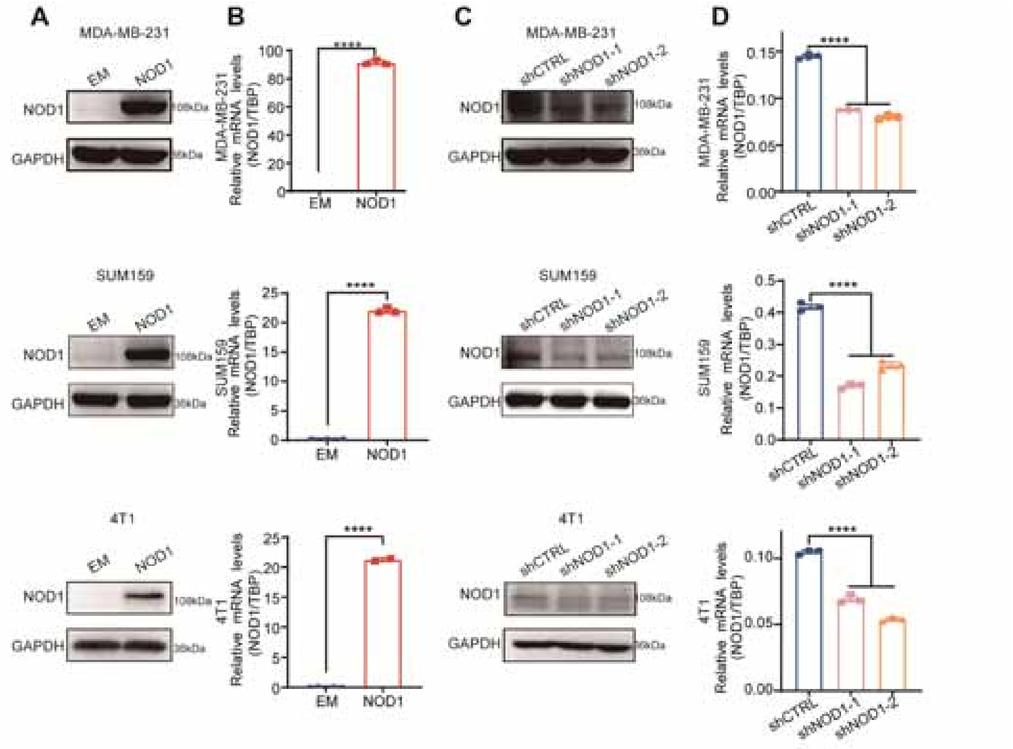

Figure. S6 The efficiency of NOD1 overexpressing or knockdown in breast cancer cells.

(A) The protein level of NOD1 was detected by western blotting in EM or NOD1-overexpressing breast cancer cells.

(B) The mRNA expression level of NOD1 was detected by qRT-PCR in EM or NOD1-overexpressing breast cancer cells. The bar graph was presented as mean ± SEM of three biological independent experiments, **** $p < 0.0001$.

(C) The protein level of NOD1 was detected by western blotting in shCTRL or NOD1-knockdown (shNOD1-1, shNOD1-2) breast cancer cells.

(D) The mRNA expression level of NOD1 was detected by qRT-PCR in shCTRL or NOD1-knockdown (shNOD1-1, shNOD1-2) breast cancer cells. The bar graph was presented as mean ± SEM of three biological independent experiments, **** $p < 0.0001$.

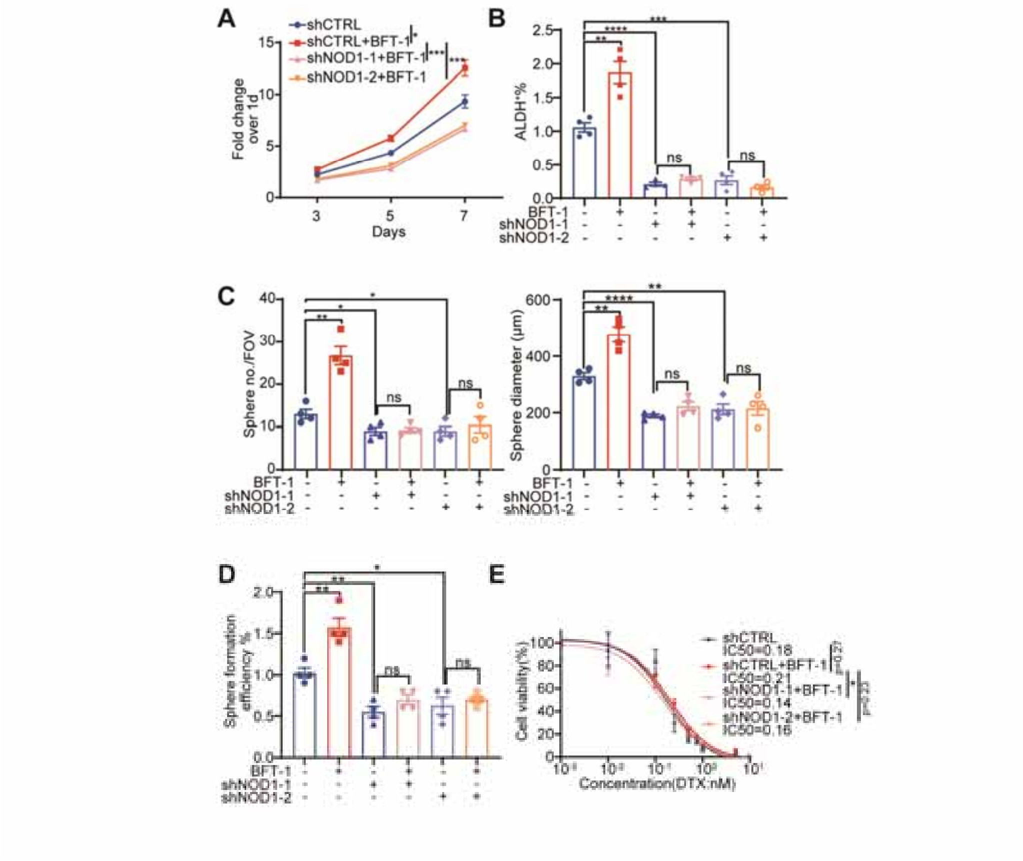

Figure. S7 NOD1 mediated BFT-1-enhanced cancer cell stemness and chemoresistance.

(A) The cell proliferation was detected by MTT assay in the control (shCTRL) or NOD1-knockdown (shNOD1-1, shNOD1-2) MDA-MB-231 cells treated with or without 10 nM BFT-1. All values between both groups were presented as mean ± SEM, * $p< 0.05$, *** $p< 0.001$.

(B) The percentage of ALDH^{+} cells was detected by ALDEFLUOR assay in the control (shCTRL) or NOD1-knockdown (shNOD1-1, shNOD1-2) MDA-MB-231 cells treated with or without 10 nM BFT-1. The bar graph was presented as mean ± SEM of three biological independent experiments, ** $p< 0.01$, *** $p< 0.001$, **** $p< 0.0001$.

(C) The self-renewal ability was determined by primary mammosphere formation assay (sphere number and sphere diameter) in the control (shCTRL) or NOD1-knockdown (shNOD1-1, shNOD1-2) MDA-MB-231 cells treated with or without 10 nM BFT-1. The bar graph was presented as mean ± SEM of three biological independent experiments, * $p< 0.05$, ** $p< 0.01$, ns no significance.

(D) The self-renewal ability was determined by secondary mammosphere formation

(sphere formation efficiency) assay in the control (shCTRL) or NOD1-knockdown (shNOD1-1, shNOD1-2) MDA-MB-231 cells treated with or without 10 nM BFT-1. The bar graph was presented as mean ± SEM of three biological independent experiments, * $p < 0.05$, ** $p < 0.01$, ns no significance.

(E) Half maximal inhibitory concentration (IC50) values of Docetaxel (DTX) were evaluated by MTT assay in shCTRL or NOD1-knockdown (shNOD1-1, shNOD1-2) MDA-MB-231 cells treated with or without 10 nM BFT-1. All values were presented as mean ± SEM, * $p < 0.05$.

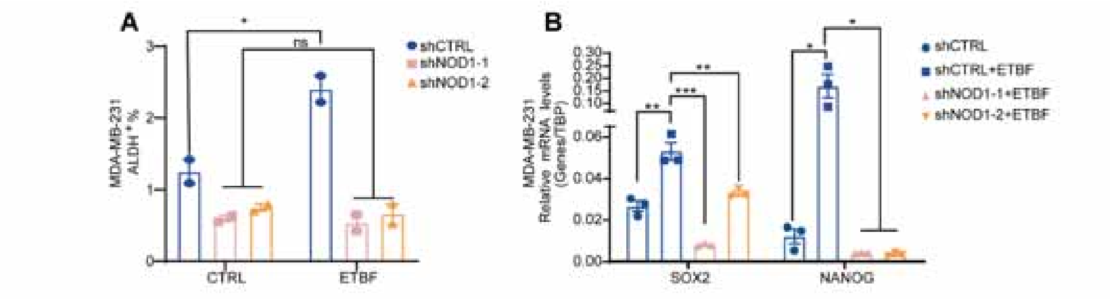

Figure. S8 NOD1 mediates ETBF-enhanced cancer cell stemness.

(A) The percentage of $ALDH^+$ cells was detected by ALDEFLUOR assay in shCTRL or NOD1-knockdown (shNOD1-1, shNOD1-2) MDA-MB-231 cells treated with or without ETBF. The bar graph was presented as mean ± SEM of three biological independent experiments, * $p< 0.05$, ns no significance.

(B) The mRNA expression levels of stemness genes SOX2, OCT4 and NANOG were detected by qRT-PCR in shCTRL or NOD1-knockdown (shNOD1-1, shNOD1-2) MDA-MB-231 cells treated with or without ETBF. The bar graph was presented as mean ± SEM, * $p< 0.05$, ** $p< 0.01$, *** $p< 0.001$.

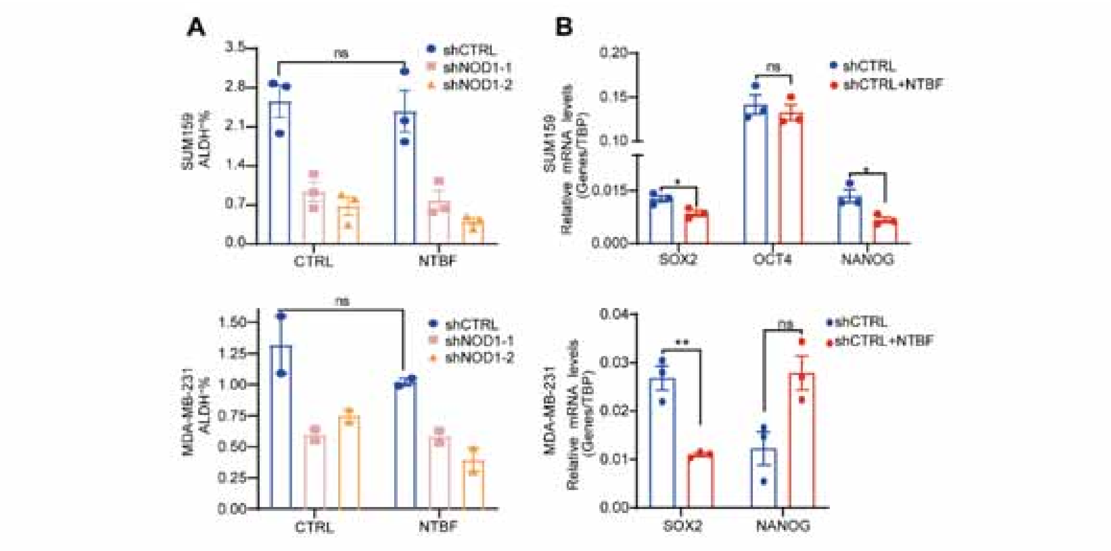

Figure. S9 Non-toxigenic *Bacteroides fragilis* (NTBF) has no effects on breast cancer cell stemness.

(A) The percentage of $ALDH^+$ cells was detected by ALDEFLUOR assay in shCTRL or NOD1-knockdown (shNOD1-1, shNOD1-2) breast cancer cells treated with or without NTBF. The bar graph was presented as mean ± SEM of three biological independent experiments, ns no significance.

(B) The mRNA expression levels of stemness genes SOX2, OCT4 and NANOG were detected by qRT-PCR in shCTRL or NOD1-knockdown (shNOD1-1, shNOD1-2) breast cancer cells treated with or without NTBF. The bar graph was presented as mean ± SEM, * $p < 0.05$, ** $p < 0.01$, ns no significance.

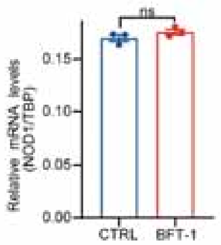

Figure. S10 The mRNA expression level of NOD1 was not affected by BFT-1 in breast cancer cells.

The mRNA expression levels of NOD1 were detected by qRT-PCR in MDA-MB-231 cells treated with or without 10 nM BFT-1. The bar graph was presented as mean ± SEM of three biological independent experiments, ns no significance.

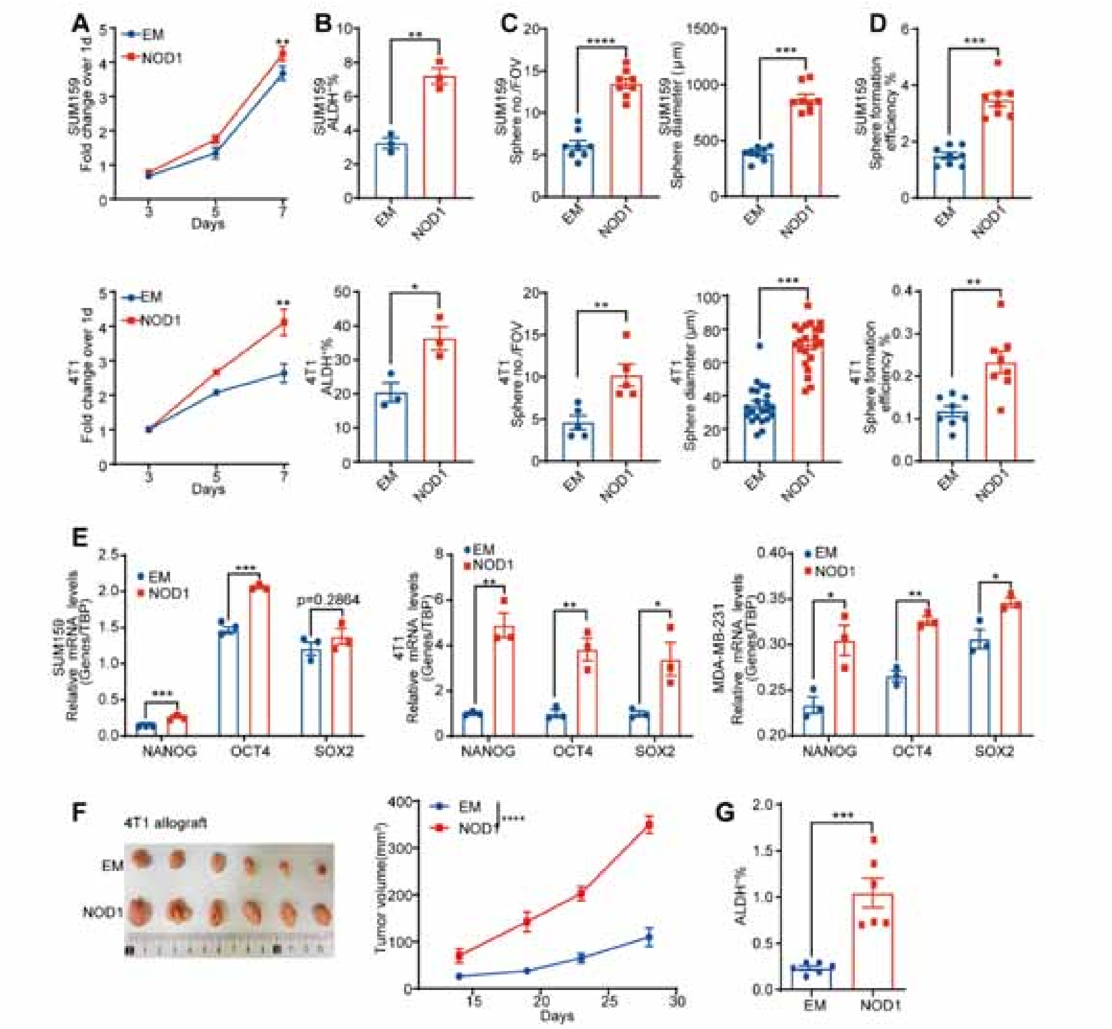

Figure. S11 NOD1 promotes cancer cell stemness and chemoresistance.

(A) The cell proliferation was analyzed by MTT assay in EM or NOD1-overexpressing breast cancer cells All values between both groups were presented as mean ± SEM, ** $p < 0.01$.

(B) The percentage of $ALDH^+$ cells was detected by ALDEFLUOR assay in EM or NOD1-overexpressing breast cancer cells. The bar graph was presented as mean ± SEM of three biological independent experiments, ** $p < 0.01$.

(C) The self-renewal ability was determined by primary mammosphere formation assay (sphere number and diameter) in EM or NOD1-overexpressing breast cancer cells. The bar graph was presented as mean ± SEM of three biological independent experiments, ** $p < 0.01$, *** $p < 0.001$, **** $p < 0.0001$.

(D) The self-renewal ability was determined by secondary mammosphere formation assay (sphere formation efficiency) in EM or NOD1-overexpressing breast cancer cells. The bar graph was presented as mean ± SEM of three biological independent

experiments, ** $p< 0.01$, *** $p< 0.001$.

(E) The mRNA expression levels of stemness genes SOX2, OCT4 and NANOG were detected by qRT-PCR in EM or NOD1-overexpressing breast cancer cells. The bar graph was presented as mean ± SEM, * $p< 0.05$, ** $p< 0.01$, *** $p< 0.001$, ns no significance.

(F) $3x10^4$ EM or NOD1-overexpressing 4T1 cells were injected at the fourth mammary fat pads of Balb/c mice (n=6 for each group) and the tumor size was measured every four days. At the end of the experiments, the mice were sacrificed, and the tumor images and the tumor growth curve were shown. All values were presented as mean ± SEM, *** $p < 0.001$, vs the EM.

(G) The percentage of $ALDH^+$ cells was detected by ALDEFLUOR assay in tumor cells from EM or NOD1-overexpressing 4T1 cell allograft tumors. The bar graph was presented as mean ± SEM, *** $p< 0.001$.

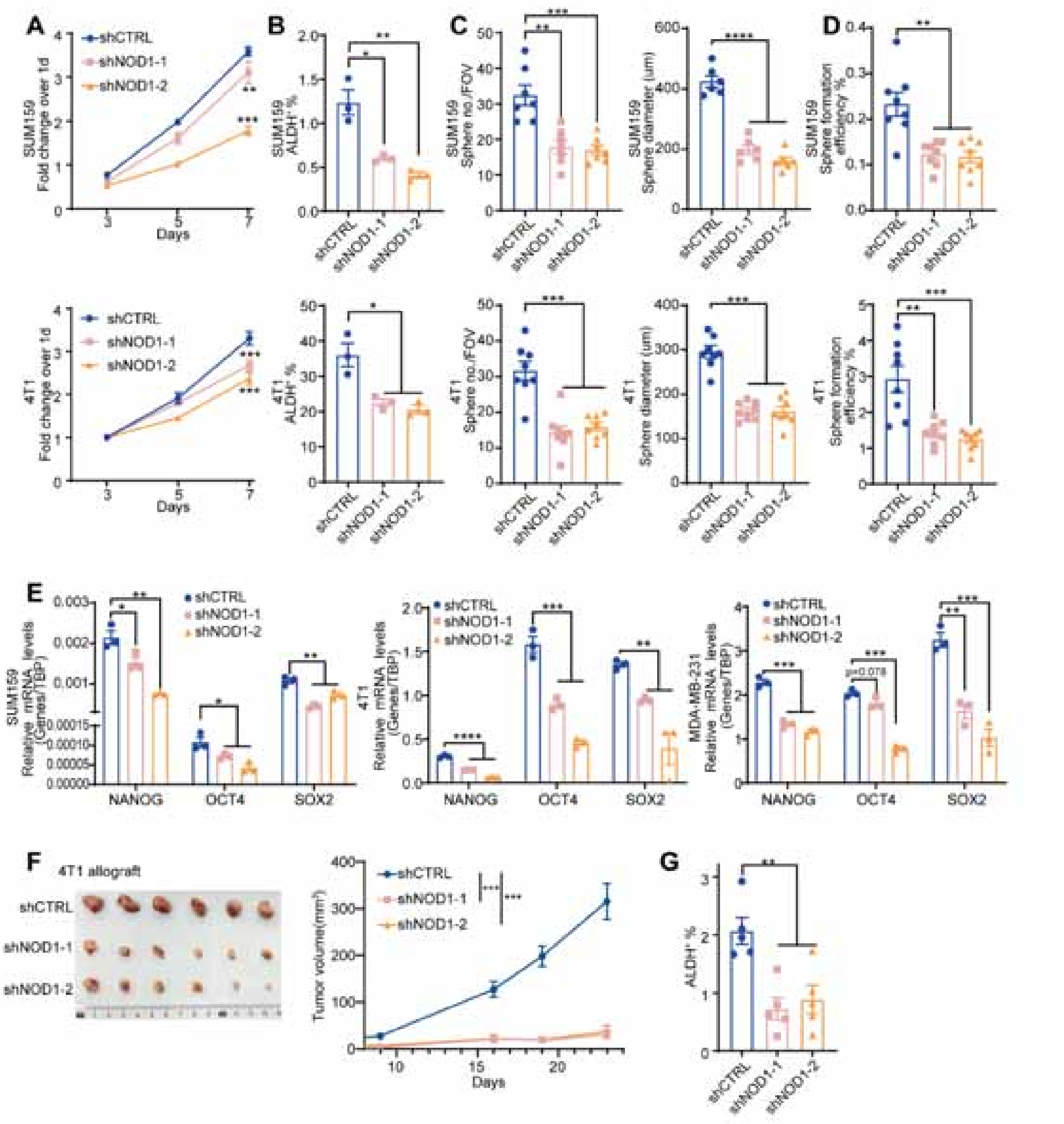

Figure. S12 NOD1 knockdown inhibits cancer cell stemness and tumor malignancy.

(A) The cell proliferation was detected by MTT assay in shCTRL or NOD1-knockdown (shNOD1-1, shNOD1-2) breast cancer cells. All values between both groups were presented as mean ± SEM, ** $p< 0.01$, *** $p< 0.001$.

(B) The percentage of $ALDH^+$ cells was detected by ALDEFLUOR assay in shCTRL or NOD1-knockdown (shNOD1-1, shNOD1-2) breast cancer cells. The bar graph was presented as mean ± SEM of three biological independent experiments, * $p< 0.05$, ** $p< 0.01$.

(C) The self-renewal ability was determined by primary mammosphere formation assay (sphere number and diameter) in shCTRL or NOD1-knockdown (shNOD1-1, shNOD1-2) breast cancer cells. The bar graph was presented as mean ± SEM of three

biological independent experiments, ** $p < 0.01$, *** $p < 0.001$, **** $p < 0.0001$.

(D) The self-renewal ability was determined by secondary mammosphere formation assay (sphere formation efficiency) in shCTRL or NOD1-knockdown (shNOD1-1, shNOD1-2) breast cancer cells. The bar graph was presented as mean ± SEM of three biological independent experiments, ** $p < 0.01$, *** $p < 0.001$.

(E) The mRNA was isolated in shCTRL or NOD1-knockdown (shNOD1-1, shNOD1-2) breast cancer cells. The mRNA expression levels of stemness genes SOX2, OCT4 and NANOG were detected by qRT-PCR. The bar graph was presented as mean ± SEM, * $p < 0.05$, ** $p < 0.01$, *** $p < 0.001$, **** $p < 0.0001$, ns no significance.

(F) 3×10^4 shCTRL or NOD1-knockdown (shNOD1-1, shNOD1-2) 4T1 cells were injected at the fourth mammary fat pads of Balb/c mice (n=6 for each group). At the end of the experiments, the mice were sacrificed, and the tumor images and the tumor growth curve were shown. All values were presented as mean ± SEM, *** $p < 0.001$, vs the shCTRL.

(G) The percentage of $ALDH^+$ cells was detected by ALDEFLUOR assay in tumor cells from shCTRL or NOD1-knockdown (shNOD1-1, shNOD1-2) 4T1 cell allograft tumors. The bar graph was presented as mean ± SEM, * $p < 0.05$.

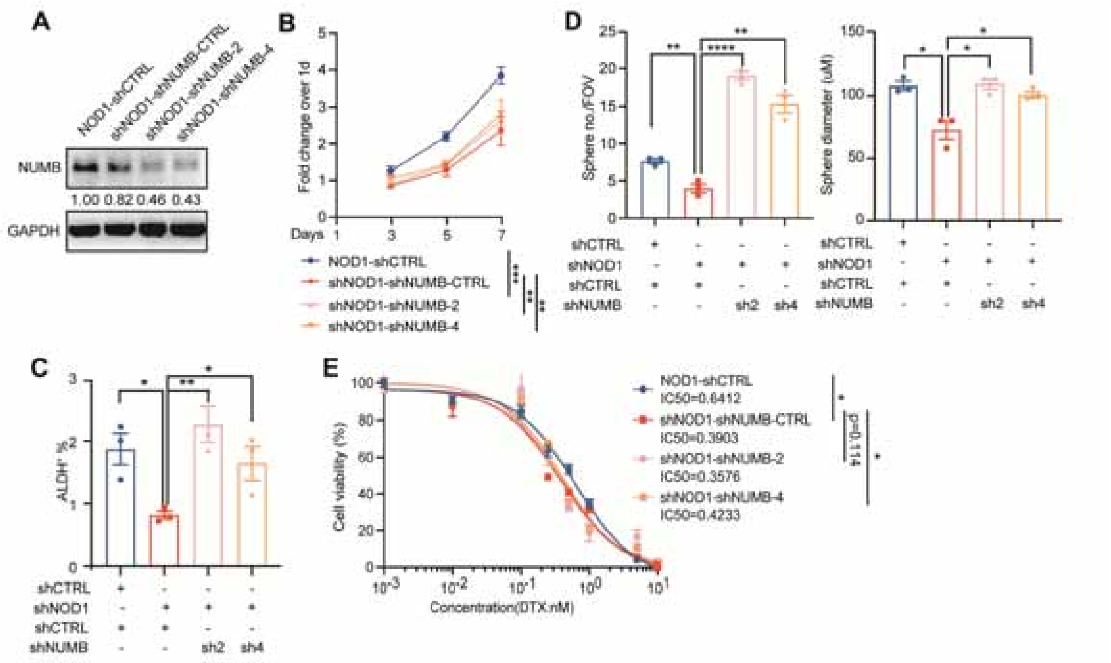

Figure. S13 Knockdown NUMB reverses the phenotype induced by knockdown NOD1.

(A) The protein level of NUMB was detected by western blotting in the control (NOD1-shCTRL), NOD1-knockdown (shNOD1-NUMB-shCTRL), or NOD1/NUMB double knockdown (shNOD1-shNUMB-2 or shNOD1-shNUMB-4) MDA-MB-231 cells.

(B) The cell proliferation was detected by MTT assay in the control (NOD1-shCTRL), NOD1-knockdown (shNOD1-NUMB-shCTRL), or NOD1/NUMB double knockdown (shNOD1-shNUMB-2 or shNOD1-shNUMB-4) MDA-MB-231 cells. All values between both groups were presented as mean ± SEM, ** $p< 0.01$.

(C) The percentage of $ALDH^{+}$ cells was detected by ALDEFLUOR assay in MDA-MB-231 cells with NOD1-knockdown or NOD1/NUMB double knockdown. The bar graph was presented as mean ± SEM of three biological independent experiments, * $p< 0.05$, ** $p< 0.01$, *** $p< 0.001$.

(D) The self-renewal ability was determined by mammosphere formation assay (sphere number and sphere diameter) in MDA-MB-231 cells with NOD1-knockdown or NOD1/NUMB double knockdown. The bar graph was presented as mean ± SEM of three biological independent experiments, * $p< 0.05$, ** $p< 0.01$, **** $p< 0.0001$.

(E) Half maximal inhibitory concentration (IC50) values of Docetaxel (DTX) were evaluated by MTT assay in the control, NOD1-knockdown, or NOD1/NUMB double knockdown MDA-MB-231 cells. All values were presented as mean ± SEM, * $p<$

0.05.

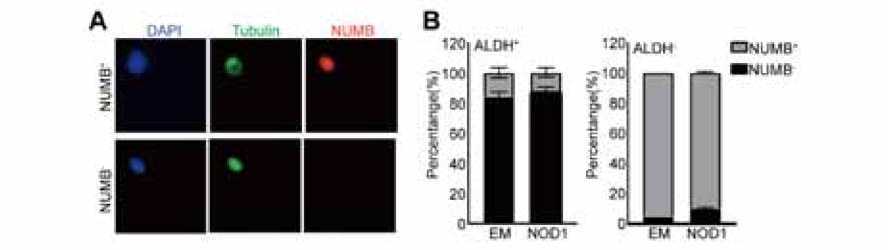

Figure. S14 NUMB expression level is shown in ALDH$^-$ or ALDH$^+$ cells.

(A) Representative images of NUMB$^-$ or NUMB$^+$ cells were shown by immunofluorescence staining.

(B) The percentages of NUMB$^+$ and NUMB$^-$ cells in ALDH$^-$ or ALDH$^+$ cells were shown. Sorted ALDH$^+$ or ALDH$^-$ cells from EM or NOD1-overexpressing SUM159 cells were plated at one cell per well in 96-well plate. After 18 hours, single cells were stained for DAPI (blue), Tubulin (green) and NUMB (red). One representative result of three independent experiments with cells was shown in the bar graph.

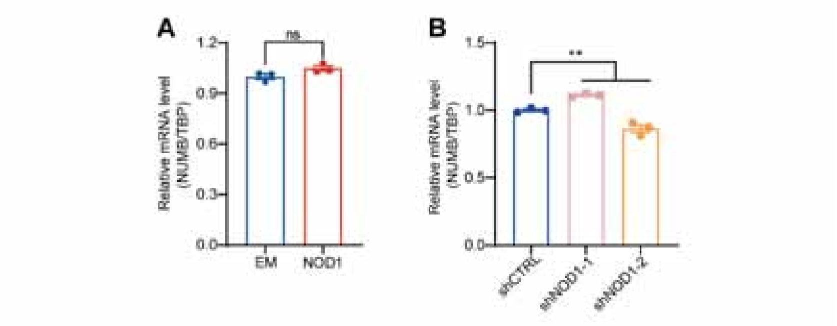

Figure. S15 The mRNA expression level of NUMB was not affected by NOD1 in breast cancer cells.

(A) The mRNA expression level of NUMB was detected by qRT-PCR in EM or NOD1-overexpressing SUM159 cells. The bar graph was presented as mean ± SEM of three biological independent experiments, ns no significance.

(B) The mRNA expression level of NUMB was detected by qRT-PCR in shCTRL or NOD1-knockdown (shNOD1-1, shNOD1-2) SUM159 cells. The bar graph was presented as mean ± SEM of three biological independent experiments, ** $p< 0.01$.

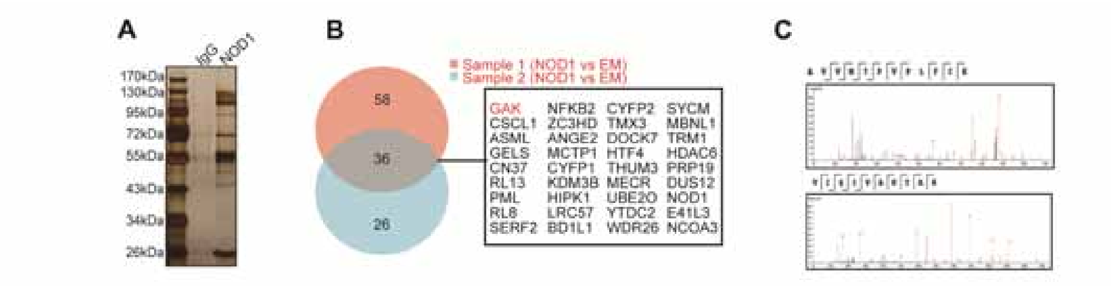

Figure. S16 Identification of NOD1-binding proteins.

(A) A representative image of silver staining after Co-Immunoprecipitation was shown in FLAG-tagged NOD1-overexpressing SUM159 cells.

(B) Candidate proteins interacting with NOD1 were identified by a liquid chromatography-tandem mass spectrometry (LC/MS) analysis.

(C) GAK peptides were identified in the Co-Immunoprecipitation samples of FLAG-tagged NOD1-overexpressing SUM159 cells by a LC/MS analysis.

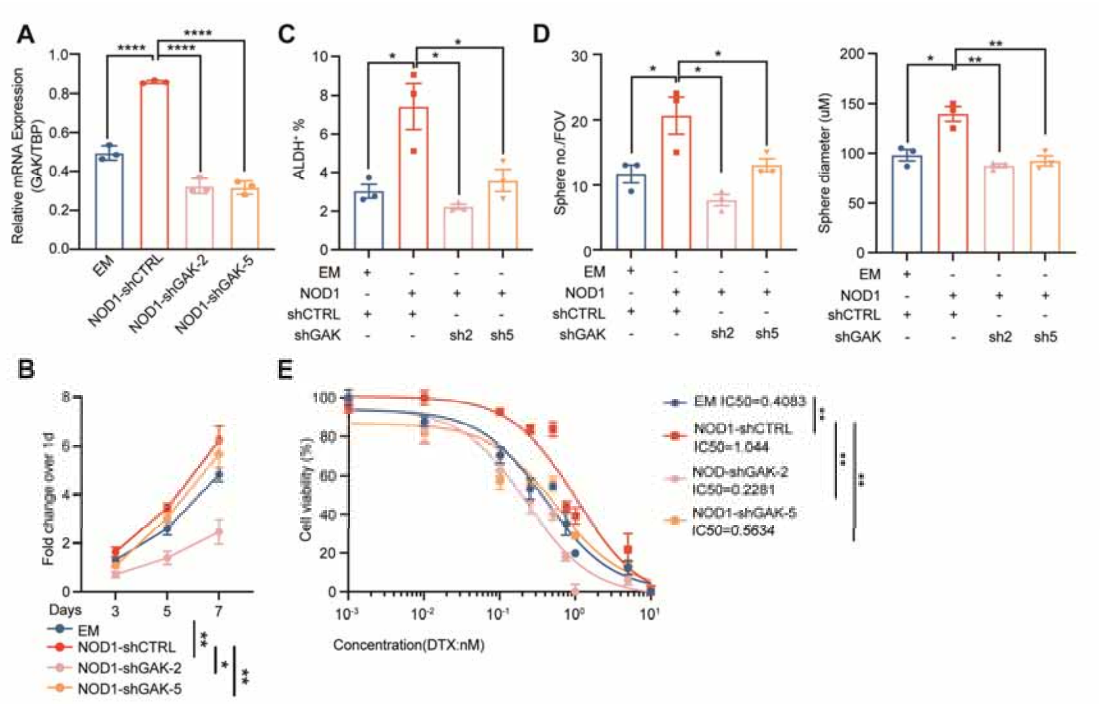

Figure. S17 Knockdown GAK reversed the phenotype induced by NOD1 overexpression.

(A) The mRNA expression level of GAK was detected by qRT-PCR in the EM or NOD1-overexpressing or NOD1-overexpressing/GAK-knockdown (shCTRL, NOD1-shGAK2 or NOD1-shGAK5) MDA-MB-231 cells. The bar graph was presented as mean ± SEM of three biological independent experiments, **** $p < 0.0001$.

(B) The cell proliferation was detected by MTT assay in the EM or NOD1-overexpressing or NOD1-overexpressing/GAK-knockdown (shCTRL, NOD1-shGAK2 or NOD1-shGAK5) MDA-MB-231 cells. All values between both groups were presented as mean ± SEM, * $p < 0.05$, ** $p < 0.01$.

(C) The percentage of $ALDH^+$ cells was detected by ALDEFLUOR assay. The bar graph was presented as mean ± SEM of three biological independent experiments, * $p < 0.05$.

(D) The self-renewal ability was determined by mammosphere formation assay (sphere number and sphere diameter). The bar graph was presented as mean ± SEM of three biological independent experiments, * $p < 0.05$, ** $p < 0.01$.

(E) Half maximal inhibitory concentration (IC50) values of Docetaxel (DTX) were evaluated by MTT assay in the EM or NOD1-overexpressing or NOD1-overexpressing/GAK-knockdown (shCTRL, NOD1-shGAK2 or NOD1-shGAK5)

MDA-MB-231 cells. All values were presented as mean ± SEM, ** $p < 0.01$, *** $p < 0.001$.

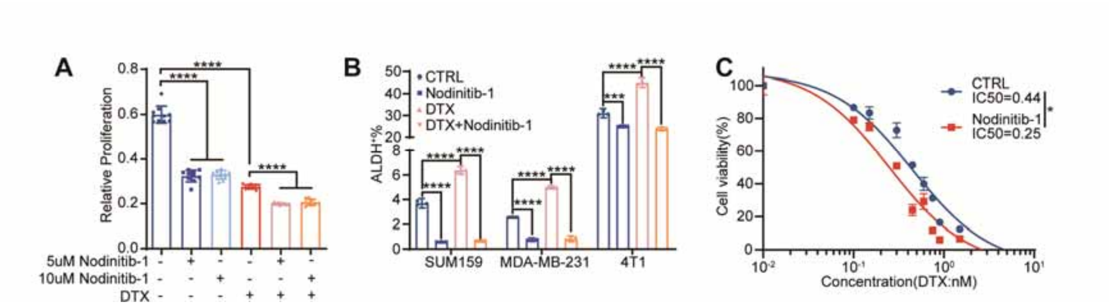

Figure. S18 Nodinitib-1 enhances chemosensitivity by targeting ALDH$^+$ BCSCs.

(A) The cell proliferation was analyzed by MTT assay in 4T1 cells treated with the NOD1 inhibitor Nodinitib-1 alone or combined with DTX. The bar graph was presented as mean ± SEM of three biological independent experiments, *** $p< 0.001$, **** $p< 0.0001$.

(B) The percentage of ALDH$^+$ cells was detected by ALDEFLUOR assay in breast cancer cells treated with Nodinitib-1 alone or combined with DTX. The bar graph was presented as mean ± SEM of three biological independent experiments, **** $p< 0.0001$.

(C) Half maximal inhibitory concentration (IC50) values of DTX were evaluated by MTT assay in SUM159 cells treated with Nodinitib-1. All values were presented as mean ± SEM, * $p< 0.05$.

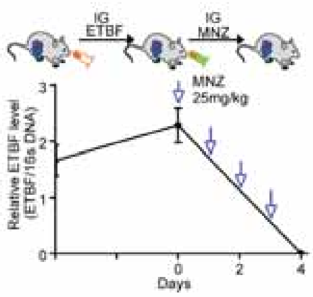

Figure. S19 MNZ efficiently eliminates ETBF in mice.

ETBF clearance efficiency was analyzed by qRT-PCR in ETBF-pretreated Balb/c mice after being treated with Metronidazole (MNZ, 25 mg/kg) by IG for consecutive four days.

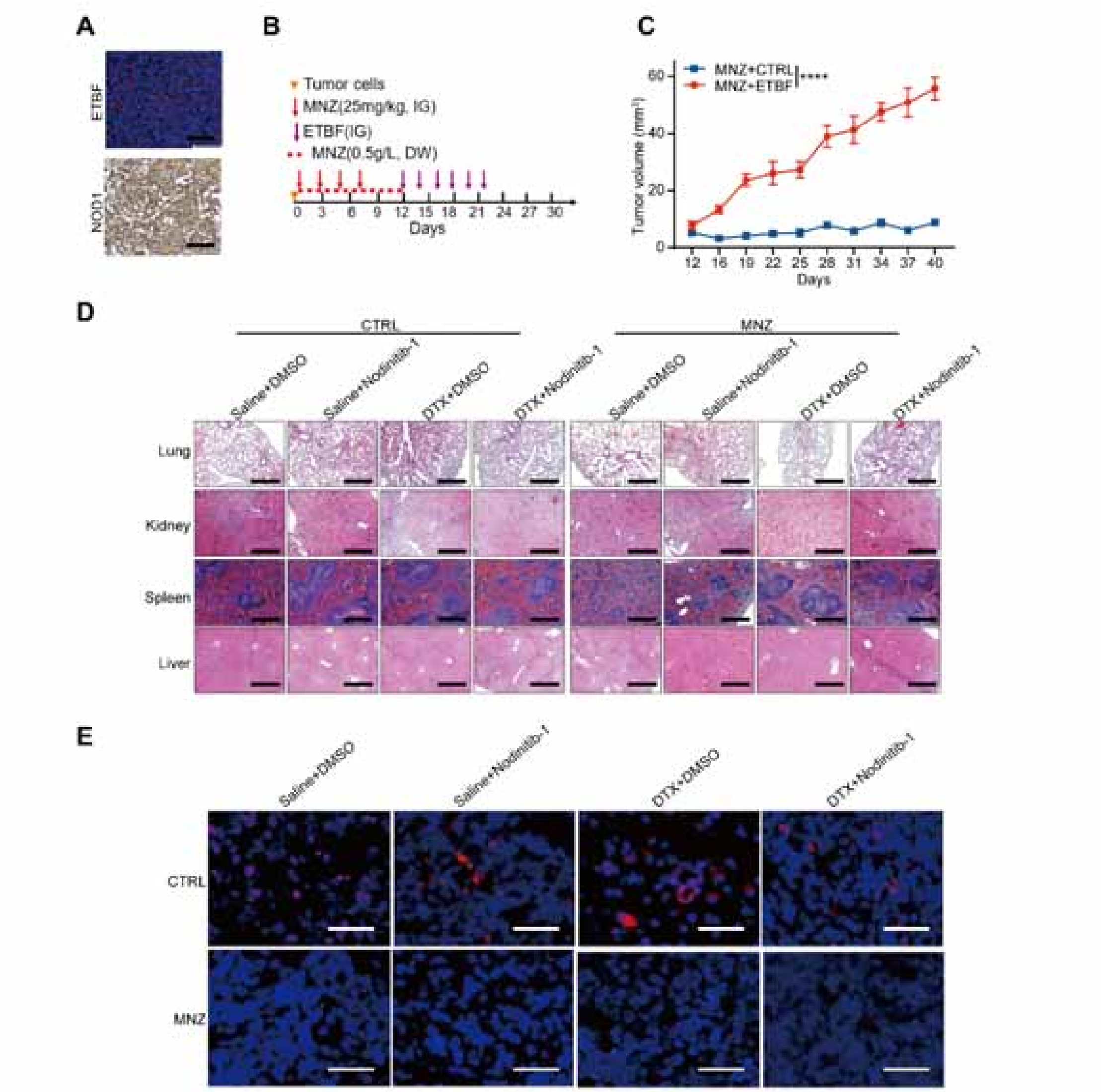

Figure. S20 Simultaneous NOD1 inhibition and ETBF clearance improve the chemosensitivity in breast cancer.

(A) ETBF abundances and NOD1 expression in PDX Lsl17 were measured by FISH and IHC respectively.

(B) Schematic design for treatment regimen. PDX Lsl17 mice were treated with MNZ (25 mg/kg) by IG every two days for four times in total and treated with MNZ (0.5 g/L) via DW throughout the experiment. ETBF ($1x10^9$ CFU) was infected via IG for six times at day 12 (n=5 for each group).

(C) The tumor size was measured every three days and the tumor growth curve were shown for PDX Lsl17 tumors in nude mice treated with ETBF (n=5). All values were presented as mean ± SEM, **** $p < 0.0001$.

(D) The Hematoxylin and Eosin (H&E) histology images were shown for different organs.

(E) ETBF clearance efficiency by MNZ in PDX Lsl17 tumors was analyzed by FISH.

Tables S1. Clinicopathological variables in the study cohort (NR/CR)

Variables	Number of patients N (%)	Response		P^a value
		NR N (%)	CR N (%)	
Total	68	27(39.71)	41(60.29)	
Age				0.4088
≤50 years	33 (48.53)	17 (25.00)	16 (23.53)	
>50 years	35 (51.47)	10 (14.71)	25 (36.76)	
Tumor size				0.5625
≤3cm	39 (57.35)	20 (29.41)	19 (27.94)	
>3cm	29 (42.65)	7 (10.29)	22 (32.35)	
Lymph node status				0.6759
Negative	11 (16.18)	5 (7.35)	6 (8.82)	
Positive	57 (83.82)	22 (32.35)	35 (51.47)	
Stage				0.0505
I	4 (5.88)	2 (2.94)	2 (2.94)	
II	50 (73.53)	17 (25.00)	33 (48.53)	
III	8 (11.76)	7 (10.29)	1 (1.47)	
Unknown	6 (8.82)	1 (1.47)	5 (7.35)	
ER status				*0.0136
Negative	45 (66.18)	15 (22.06)	30 (44.12)	
Positive	23 (47.06)	12 (17.65)	11 (16.18)	
PR status				***0.0004
Negative	49 (72.06)	16 (23.53)	33 (48.53)	
Positive	19 (27.94)	11 (16.18)	8 (11.76)	
HER2 status				0.1547
Negative	36 (52.94)	17 (25.00)	19 (27.94)	
Positive	32 (47.06)	10 (14.71)	22 (32.35)	
Subtype[b]				0.1256
Luminal	25 (36.76)	12 (17.65)	13 (19.12)	
HER-2 Enrichment	21 (30.88)	7 (10.29)	14 (20.59)	
TNBC	22 (32.35)	8 (11.76)	14 (20.59)	

Abbreviations: ER, estrogen receptor; PR, progesterone receptor; HER-2, human epidermal growth factor receptor 2; TNBC, triple negative breast cancer;

[a] Based-on Pearson $\chi2$ test (Fisher exact test was used when needed).

[b] Definition of subtypes: Luminal (ER and/or PR positive), HER-2 Enrichment (ER and PR negative, HER-2 positive), and TNBC (ER negative, PR negative, and HER-2 negative)

Tables S2. Clinicopathological variables in the study cohort (PR/RE)

Variables	Number of patients N (%)
Total	25
Lymph node status	
Negative	8 (32%)
Positive	17 (68%)
ER status	
Negative	7 (28%)
Positive	18 (72%)
PR status	
Negative	11 (44%)
Positive	14 (56%)
HER2 status	
Negative	6 (24%)
Positive	19 (76%)
Subtype	
Luminal	17 (68%)
HER-2 Enrichment	7 (28%)
TNBC	1 (4%)

Tables S3. shRNA targets

**shRNA target**	**Sequence (5'→3')**
shNOD1(h)-1	CCGGGTCCGAGTTCTTCCTCTACTTCTCGAGAAGTAGAGGAAGAACT CGGACTTTTTTG
shNOD1(h)-2	CCGGCCTAGACCTAGACAACAACAACTCGAGTTGTTGTTGTCTAGGT CTAGGTTTTTTG
shNOD1(m)-1	CCGGTGAGGAACTGACCAAGTATAACTCGAGTTATACTTGGTCAGT TCCTCATTTTTG
shNOD1(m)-2	CCGGCCCAGTGACTGCATGGTTATTCTCGAGAATAACCATGCAGTC ACTGGGTTTTTG
shGAK-2	CCGGCCAGAATTGCAGTGATGTCATCTCGAGATGACATCACTGCAA TTCTGGTTTTT
shGAK-5	CCGGGAGAACTTGTTGCTTAGTAACCTCGAGGTTACTAAGCAACAA GTTCTCTTTTTTG
shNUMB-2	GCAATCATTATGGCTATGTATCTCGAGATACATAGCCATAATGATT GCTTTTT
shNUMB-4	GCCATGTAGAAGTTGATGAATCTCGAGATTCATCAACTTCTACATG GCTTTTT

Tables S4. Primer pairs for qRT-PCR

Gene	Primer pair	Sequence (5'→3')
h TBP	Forward	TGCACAGGAGCCAAGAGTGAA
	Reverse	CACATCACAGCTCCCCACCA
h NOD1	Forward	ACTGAAAAGCAATCGGGAACTT
	Reverse	CACACACAATCTCCGCATCTT
h HEY1	Forward	TTGGCCAGAAAAAGACGG
	Reverse	ATCTGCAGGATCTCGGCTT
h NUMB	Forward	TCAGCAGATGGACTCAGAGTT
	Reverse	AGGCTCTATCAAAGTTCCTGTCT
h NANOG	Forward	AATACCTCAGCCTCCAGCAGATG
	Reverse	TGCGTCACACCATTGCTATTCTTC
h SOX2	Forward	GCACATGAACGGCTGGAGCAACG
	Reverse	TGCTGCGAGTAGGACATGCTGTAGG
h OCT4	Forward	CTGGGTTGATCCTCGGACCT
	Reverse	CACAGAACTCATACGGCGGG
m TBP	Forward	CCCCACAACTCTTCCATTCT
	Reverse	GCAGGAGTGATAGGGGTCAT
m NOD1	Forward	GAAGGCACCCCATTGGGTT
	Reverse	AATCTCTGCATCTTCGGCTGA
m NANOG	Forward	TCTTCCTGGTCCCCACAGTTT
	Reverse	GCAAGAATAGTTCTCGGGATGAA
m SOX2	Forward	GCGGAGTGGAAACTTTTGTCC
	Reverse	CGGGAAGCGTGTACTTATCCTT
m OCT4	Forward	GGCTTCAGACTTCGCCTCC
	Reverse	AACCTGAGGTCCACAGTATGC
16S	Forward	GGTGAATACGTTCCCGG
	Reverse	TACGGCTACCTTGTTACGACTT

BFT	Forward	GGATACATCAGCTGGGTTGTAG
	Reverse	GCGAACTCGGTTTATGCAGT

Note: All primers were verified for specificity via Primer-BLAST (https://www.ncbi.nlm.nih.gov/tools/primer-blast/) and tested by melt curve.

Tables S5. Antibodies

Antibodies	Source	Identifier
Anti-NOD1(h-WB)	BioLegend	Cat# 657002
Anti-NOD1(m-WB)	Santa Cruz	Cat# sc-398696
Anti-NOD1(IHC)	Abcam	Cat# ab189435
Anti-NUMB	CST	Cat# 2756S
Anti-GAK	ProteinTech	Cat# 12147-1-AP
Anti-NOTCH1(NICD1)	CST	Cat# 3608
Anti-HEY1	ProteinTech	Cat# 19929-1-AP
Anti-p-Thr	Santa Cruz	Cat# sc-5267
Anti-HIS	Abmart	Cat# M20001
Anti-GAPDH	TransGen	Cat# HC301
Anti-FLAG	Sigma Aldrich	Cat# F7425
Anti-HA	CST	Cat# 3724
